# Supplementary material for: Host ecology and phylogeny shape the temporal dynamics of social bee viromes
Source: Nat Commun. 2025 Mar 5;16:2207. doi: 10.1038/s41467-025-57314-7 (PMC11882784; doi:10.1038/s41467-025-57314-7)

Supplementary Information for:

Host ecology and phylogeny shape the temporal dynamics of social bee viromes

Vincent Doublet[1,2,@], Toby Doyle[2], Claire Carvell[3], Mark JF Brown[4], Lena Wilfert[1,2]

1. Institute of Evolutionary Ecology and Conservation Genomics, University of Ulm, 89069 Ulm, Germany
2. Centre for Ecology and Conservation, University of Exeter, Penryn TR10 9FE, UK
3. UK Centre for Ecology & Hydrology, Benson Lane, Crowmarsh Gifford, Wallingford OX10 8BB, UK
4. Department of Biological Sciences, School of Life Sciences and the Environment, Royal Holloway University of London, Egham, UK

Correspondence

Vincent Doublet, University of Ulm, Ulm, Germany.

@. vincent.bs.doublet@gmail.com

Supplementary Method 1: Primers and protocols used to differentiate the *B. terrestris*/*B. lucorum* complex and *B. hortorum*/*B. ruderatus* complex**.**

Target	Primer name	Sequence	Amplification program	Amplicon size (bp)	References
B. terrestris/ *B. lucorum* complex	BBM1IGSF<br>BBM1IGSR	GGAGCAATAATTTCAATAAATAG<br>AARTTCAAAGCACTAATCTGC	15s at 95°C<br>15s at 55°C<br>45s at 72°C<br>×38 cycles	180<br>210	R. Schmid-Hempel (pers. comm.), Manley *et al*. 2023
B. hortorum/ *B. ruderatus* complex	CYTBF<br>CYTBR	TTCAGCAATTCCATATATTGGAC<br>ATTACACCTCCTCATTTATTAGG	30s at 94°C<br>30s at 48°C<br>60s at 72°C<br>×35 cycles	~500	Ellis *et al*. 2005

References:

Ellis, J.S., Knight, M.E. & Goulson, D. Delimiting species for conservation using molecular markers: the taxonomic status of *Bombus ruderatus* (Fabricus) and *Bombus hortorum* (Linnaeus) (Hymenoptera: Apidae). *Journal of Insect Conservation* **9**, 75-83 (2005).

Manley, R. *et al.* Conservation measures or hotspots of disease transmission? Agri-environment schemes can reduce disease prevalence in pollinator communities. *Philosophical transactions of the Royal Society of London. Series B, Biological sciences* **378**, 20220004 (2023).

Supplementary Figure 1: Stack plot showing normalized viral read numbers categorized by virus orders in each meta-transcriptome library. Magenta color shows the high prominence of picornavirales in most samples. The bottom dark color represents unclassified viral species. Source data are provided as a Source Data file.

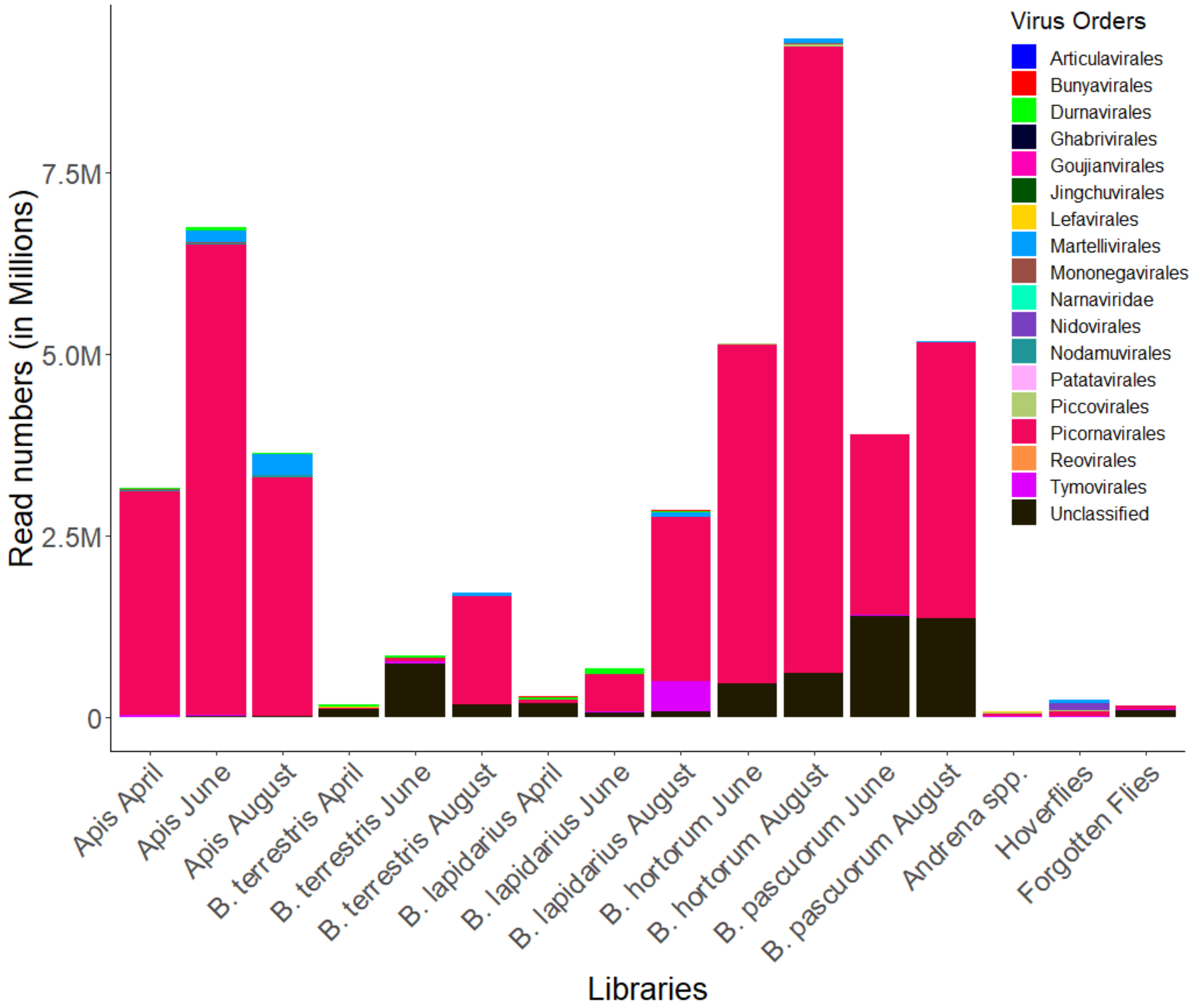

Supplementary Figure 2: Coverage plots of insect virus sequence assemblies with vsiRNA reads. Sample collection time points are represented in different colors, with April collection data in green, June collection data in red, blue collection data in blue, and mixed time point data in grey (i.e. for mining bees, hoverflies and the 'Forgotten flies'). Coverage depth is represented by number of reads (y axis) along the sequence (x axis) for each virus with detected vsiRNA reads above the defined threshold, in (2a) Apis mellifera, (2b) Bombus terrestris, (2c) Bombus lapidarius, (2d) Bombus hortorum, (2e) Bombus pascuorum, (2f) mining bees Andrena spp., (2g) hoverflies, and (2h) the 'Forgotten flies'. Abbreviations: ABPV = Acute bee paralysis virus, AhaemNLV = Andrena haemorrhoa nege-like virus, ARV = Apis rhabdovirus, BQCV = Black queen cell virus, CBPV = Chronic bee paralysis virus, DWV = Deformed wing virus, LSV = Lake Sinai virus, SBV = Sacbrood virus, SBPV = Slow bee paralysis virus, VDV-2 = Varroa descructor virus-2, VOV-1 = Varroa orthomyxovirus-1. Viral assemblies and mapping outputs are available at https://doi.org/10.6084/m9.figshare.27888378.

Supplementary Figure 2a

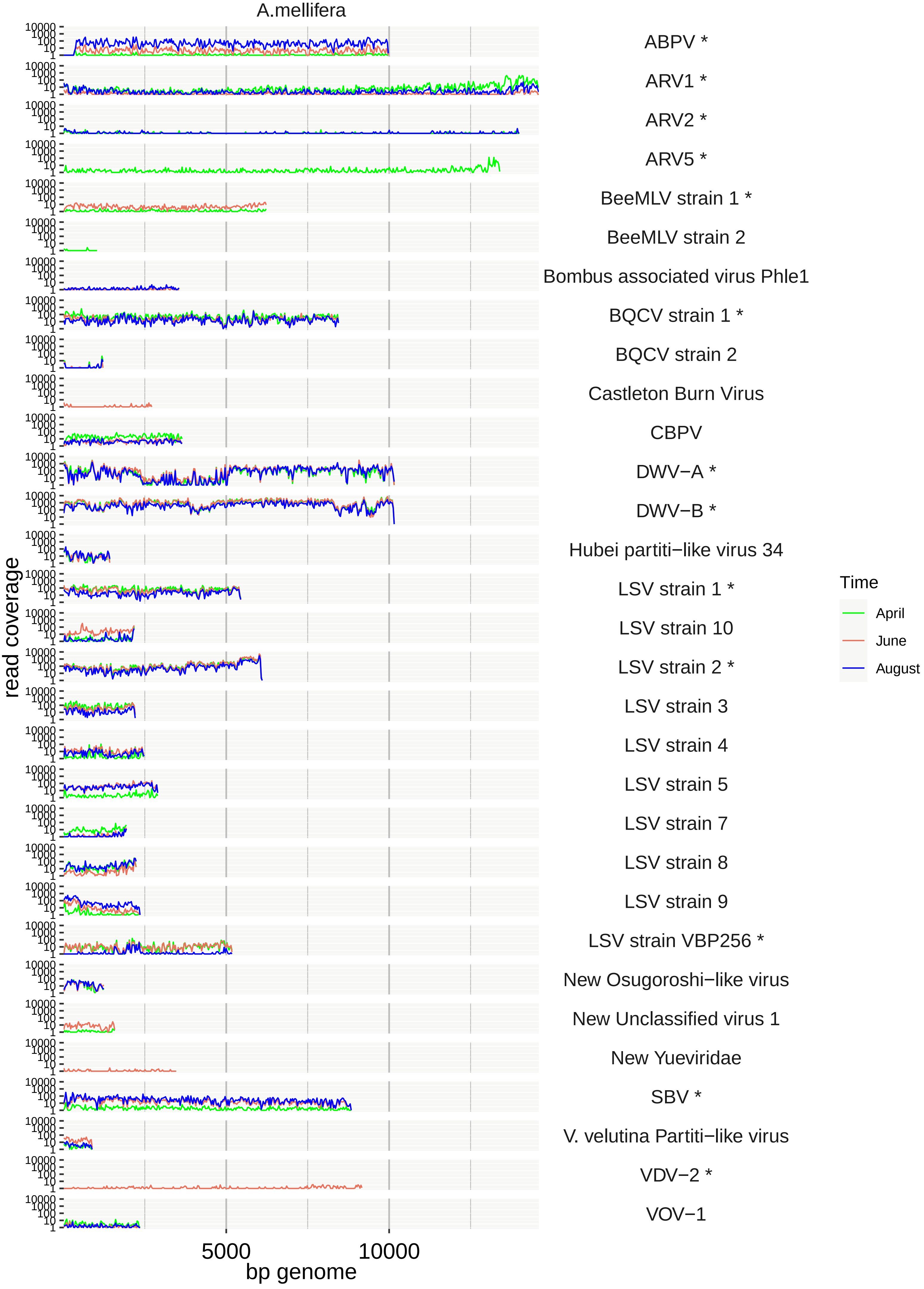

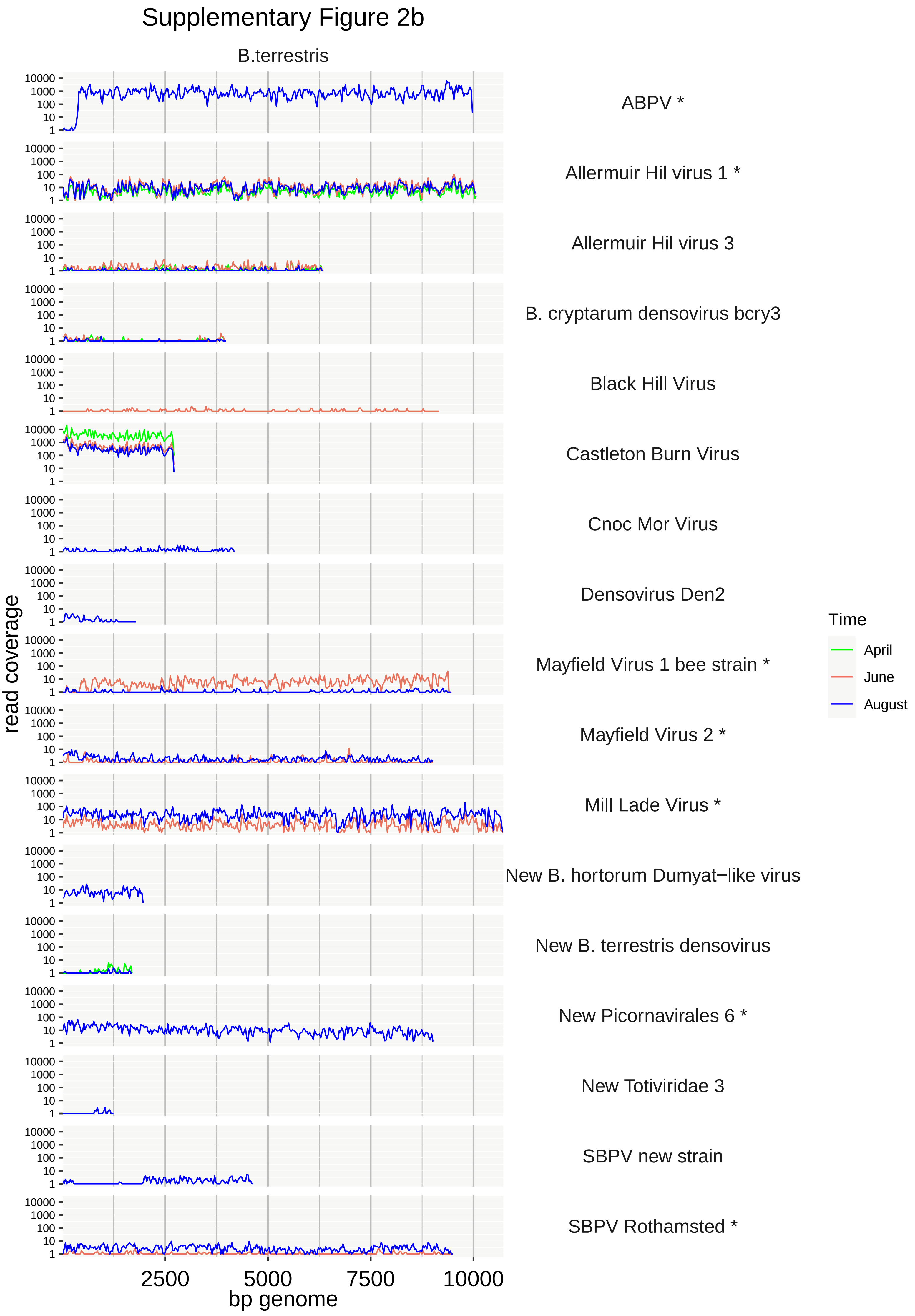
Supplementary Figure 2b
B.terrestris
ABPV *
Allermuir Hil virus 1 *
Allermuir Hil virus 3
B. cryptarum densovirus bcry3
Black Hill Virus
Castleton Burn Virus
Cnoc Mor Virus
Densovirus Den2
Mayfield Virus 1 bee strain *
Mayfield Virus 2 *
Mill Lade Virus *
New B. hortorum Dumyat−like virus
New B. terrestris densovirus
New Picornavirales 6 *
New Totiviridae 3
SBPV new strain
SBPV Rothamsted *
read coverage
bp genome
2500
5000
7500
10000
Time
April
June
August

Supplementary Figure 2c

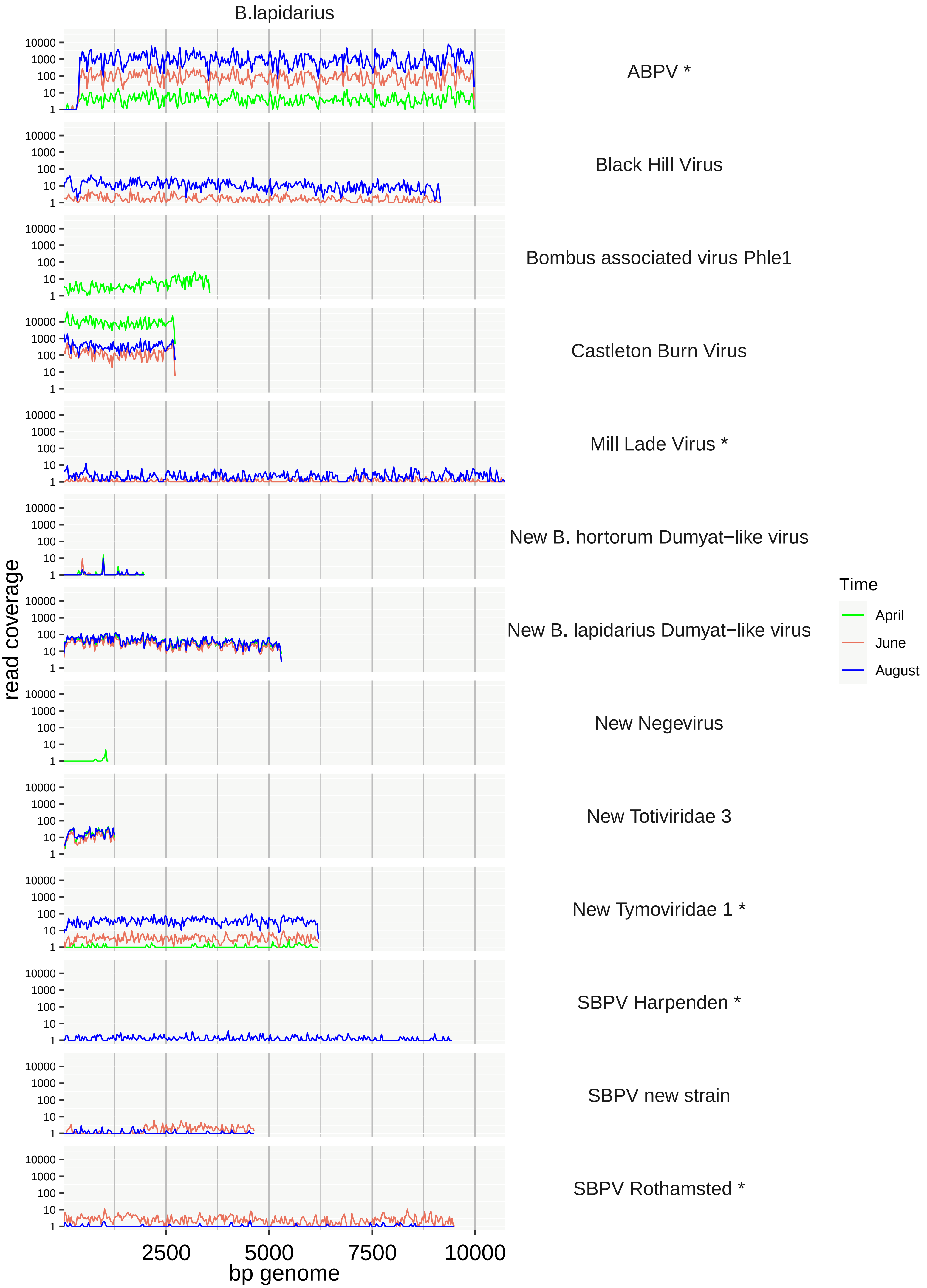

Supplementary Figure 2d

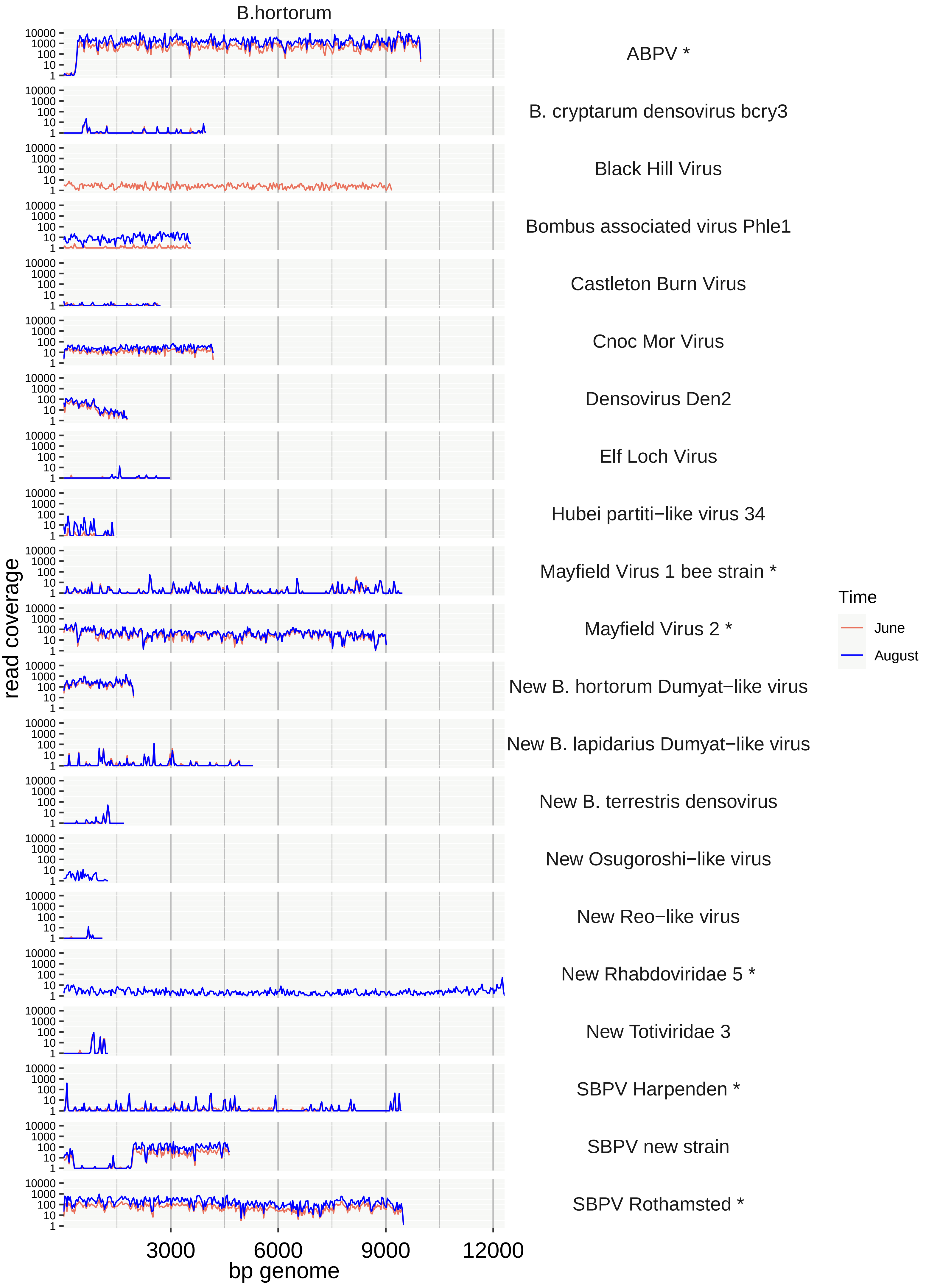

Supplementary Figure 2e

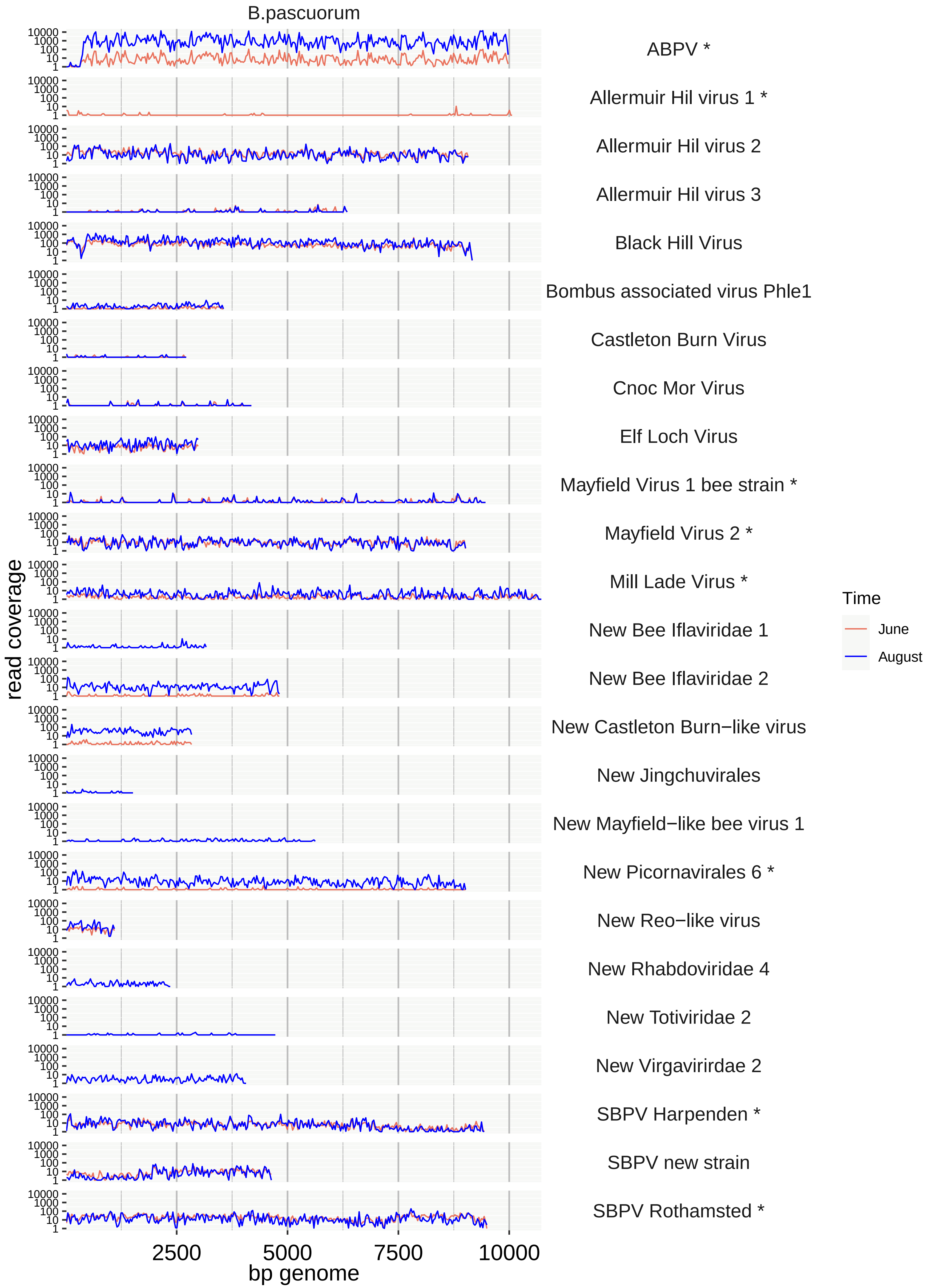

Supplementary Figure 2f

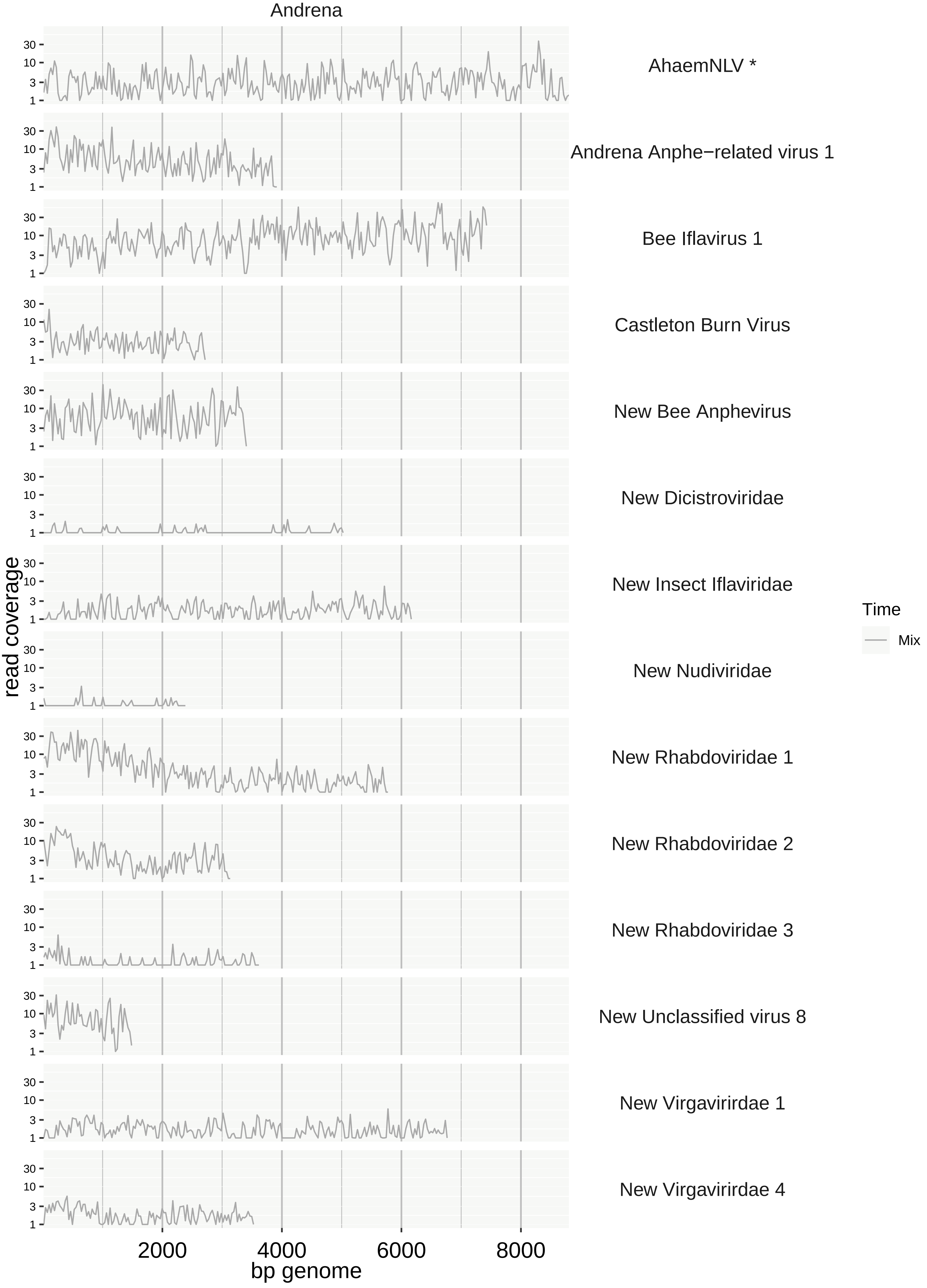

Supplementary Figure 2g

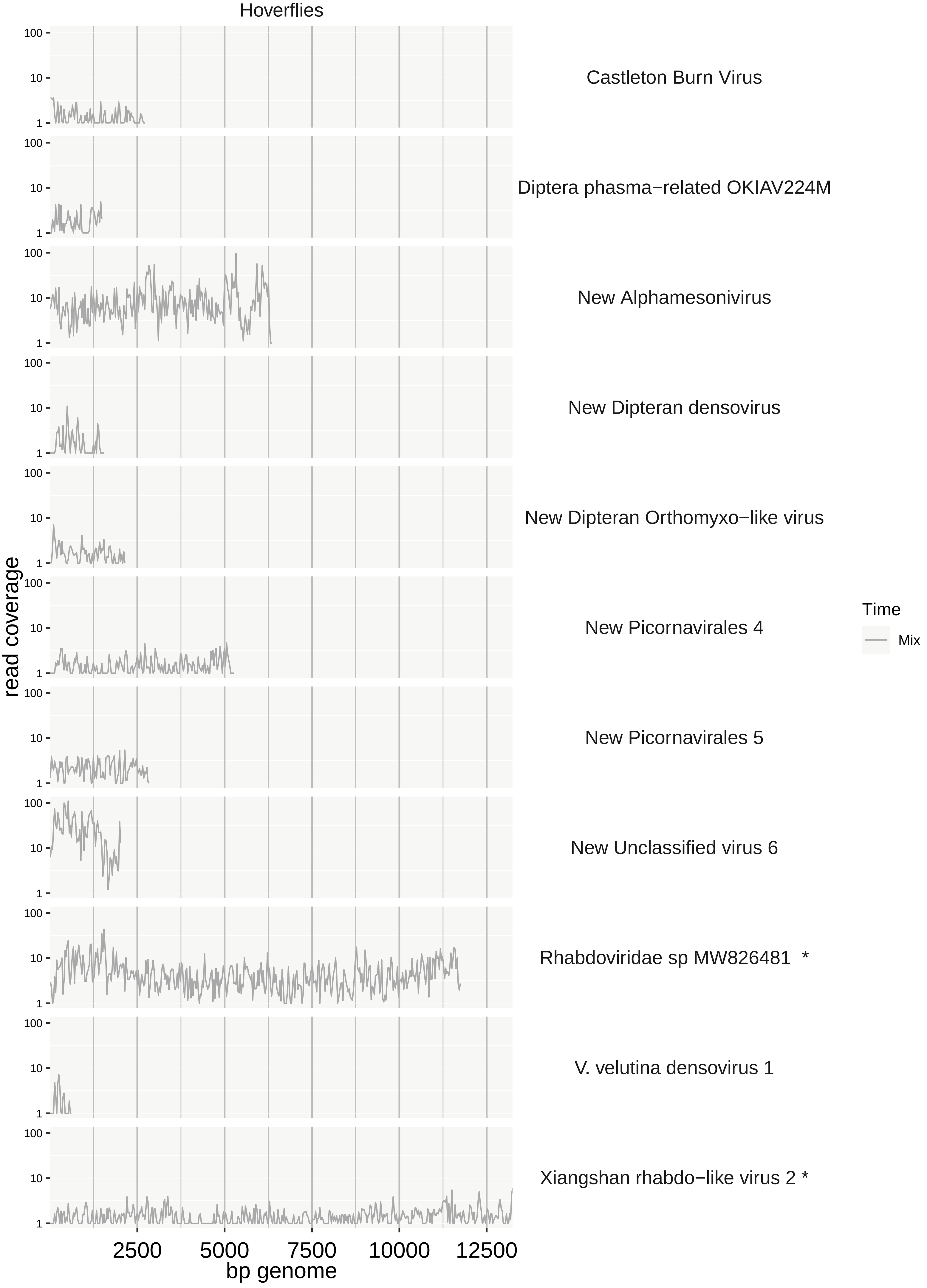

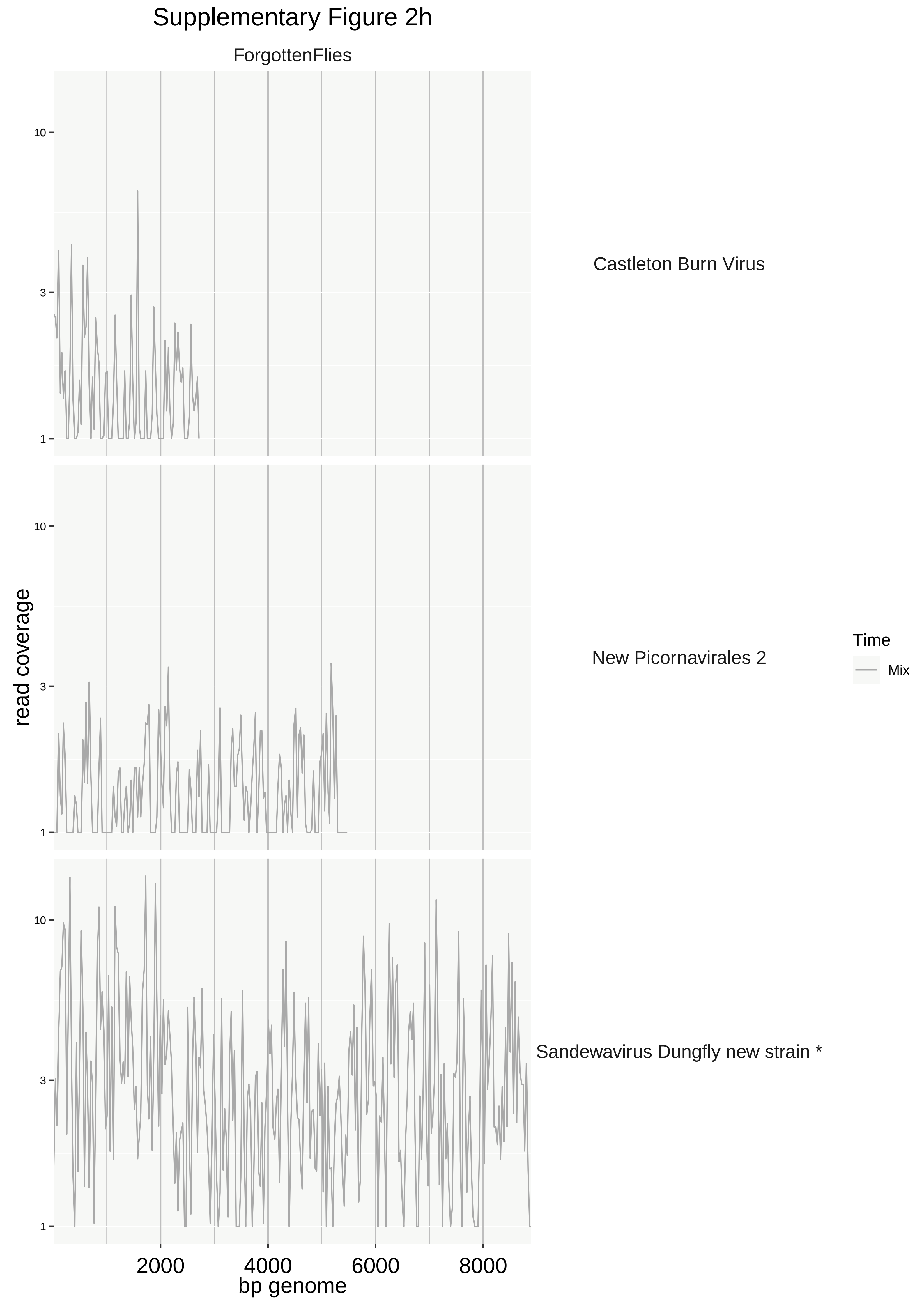

Supplementary Figure 2h
ForgottenFlies
Castleton Burn Virus
New Picornavirales 2
Sandewavirus Dungfly new strain *
read coverage
10
3
1
Time
Mix
2000
4000
6000
8000
bp genome

Supplementary Figure 3: Viral small interfering RNAs (vsiRNA) profiles showing insect and plant viruses positive and negative strand reads stacked by size, ordered by sample: (3.1 – 3.4) vsiRNA profiles from pooled *Apis mellifera* RNA from April collection, (3.5 – 3.9) vsiRNA profiles from pooled *Apis mellifera* RNA from June collection, (3.10 – 3.12) vsiRNA profiles from pooled *Apis mellifera* RNA from August collection, (3.13 – 3.14) vsiRNA profiles from pooled *Bombus terrestris* RNA from April collection, (3.15 – 3.16) vsiRNA profiles from pooled *Bombus terrestris* RNA from June collection, (3.17 – 3.19) vsiRNA profiles from pooled *Bombus terrestris* RNA from August collection, (3.20 – 3.22) vsiRNA profiles from pooled *Bombus lapidarius* RNA from April collection, (3.23 – 3.24) vsiRNA profiles from pooled *Bombus lapidarius* RNA from June collection, (3.25 – 3.26) vsiRNA profiles from pooled *Bombus lapidarius* RNA from April collection, (3.27 – 3.30) vsiRNA profiles from pooled *Bombus hortorum* RNA from June collection, (3.31 – 3.33) vsiRNA profiles from pooled *Bombus hortorum* RNA from August collection, (3.34 – 3.37) vsiRNA profiles from pooled *Bombus pascuorum* RNA from June collection, (3.38 – 3.40) vsiRNA profiles from pooled *Bombus pascuorum* RNA from August collection, (3.41 – 3.43) vsiRNA profiles from pooled mining bees (*Andrena spp.*) RNA, (3.44 – 3.45) vsiRNA profiles from pooled hoverflies RNA, and (3.46) vsiRNA profiles from pooled 'Forgotten flies' RNA. Abbreviations: ABPV = Acute bee paralysis virus, AhaemNLV = *Andrena haemorrhoa* nege-like virus, ArMV = *Arabis* mosaic virus, ARV = *Apis* rhabdovirus, BQCV = Black queen cell virus, CBPV = Chronic bee paralysis virus, CLRV = Cherry leaf roll virus, CVA = Cherry virus A, CYV = Caraway yellows virus, DWV = Deformed wing virus, LRV = *Lychnis* ringspot virus, LSV = Lake Sinai virus, PDV = *Prunus* dwarf virus, PVF = Prunus virus F, RCNVA = Red clover nepovirus A, RRV = Raspberry ringspot virus, SBV = Sacbrood virus, SBPV = Slow bee paralysis virus, VDV-2 = *Varroa descructor* virus-2, VOV-1 = *Varroa* orthomyxovirus-1. Viral assemblies and mapping outputs are available at https://doi.org/10.6084/m9.figshare.27888378.

Supplementary Figure 3.1 : *Apis mellifera* – April – insect virus vsiRNA profiles

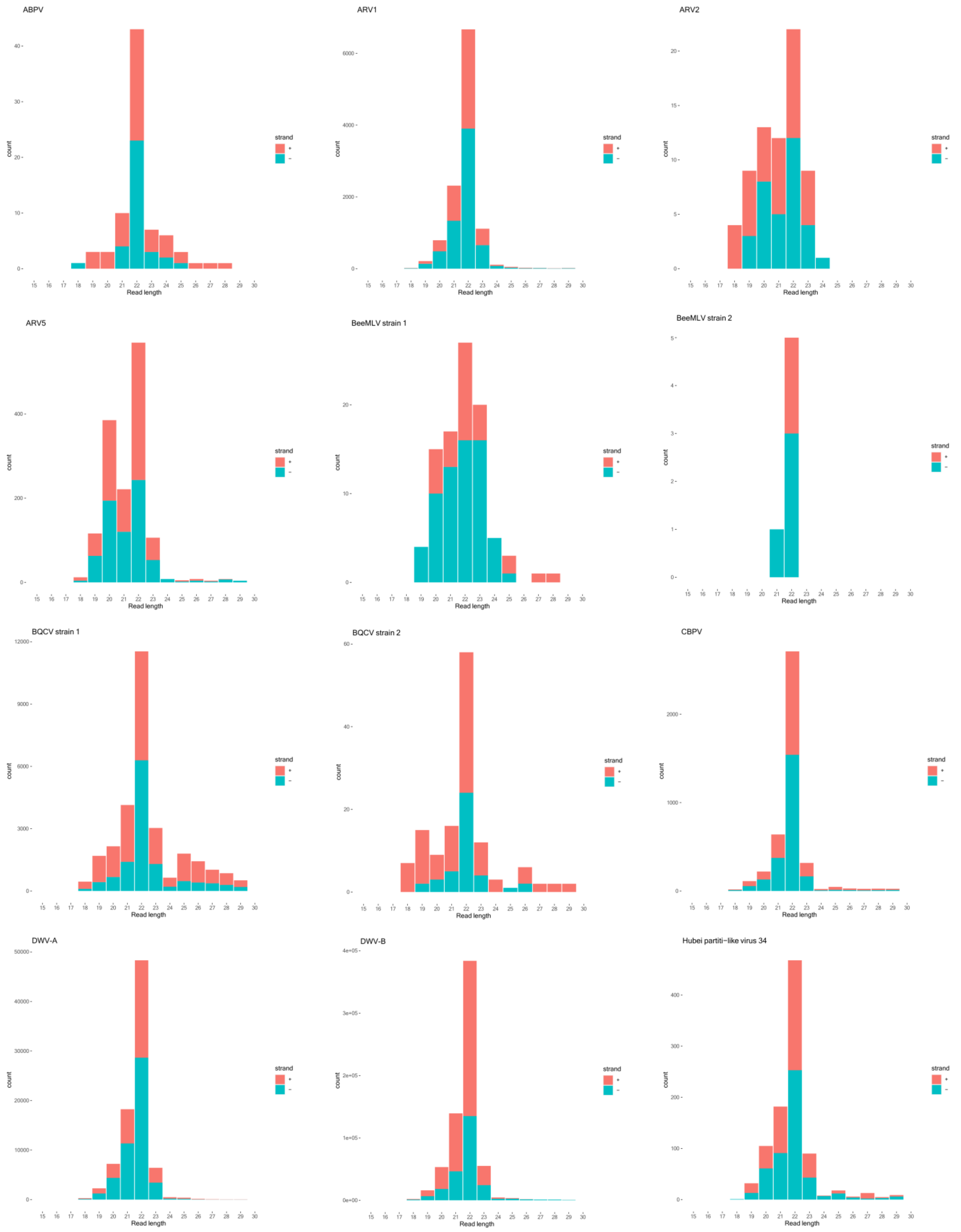

Supplementary Figure 3.2 : *Apis mellifera* – April – insect virus vsiRNA profiles

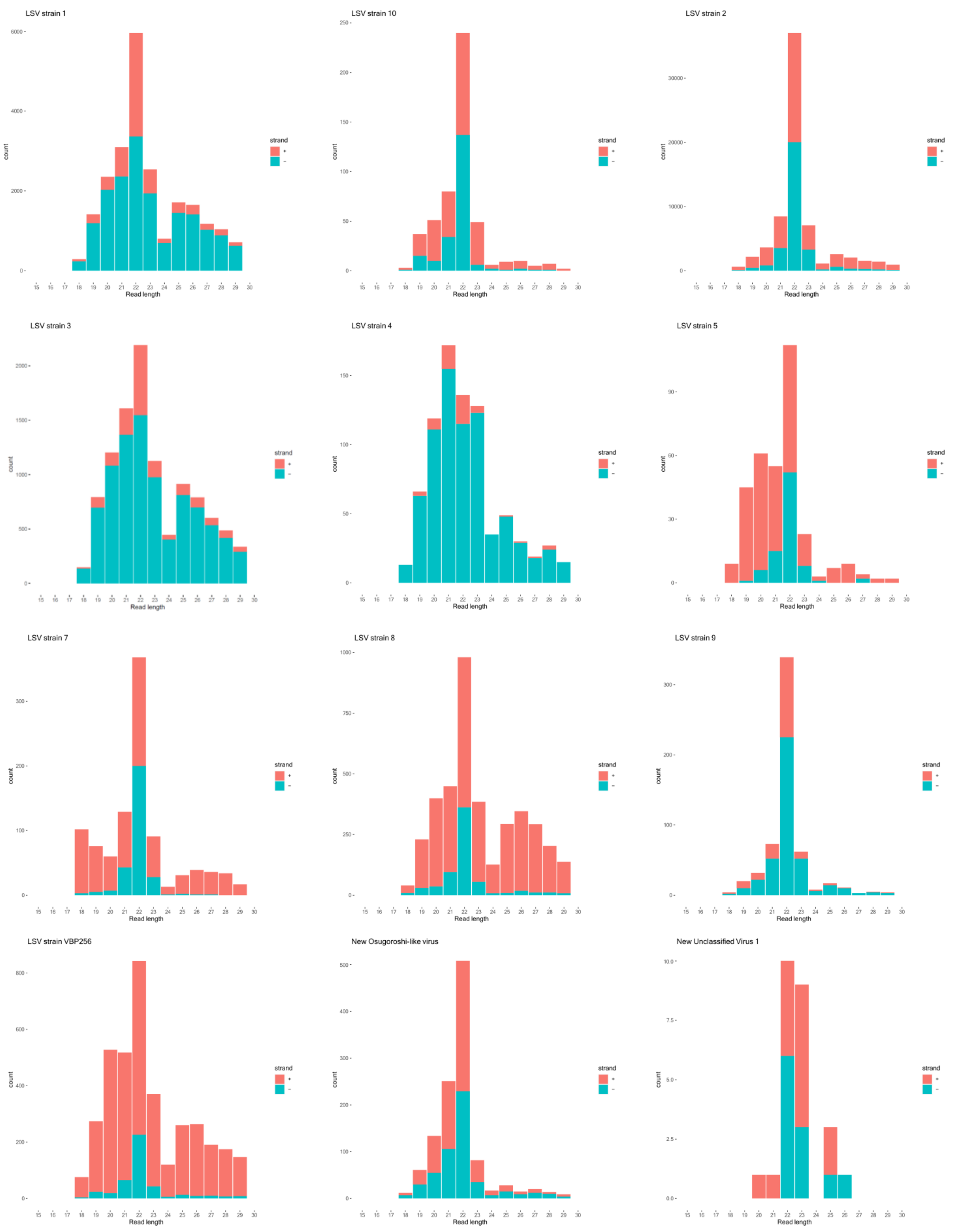

Supplementary Figure 3.3 : *Apis mellifera* – April – insect virus vsiRNA profiles

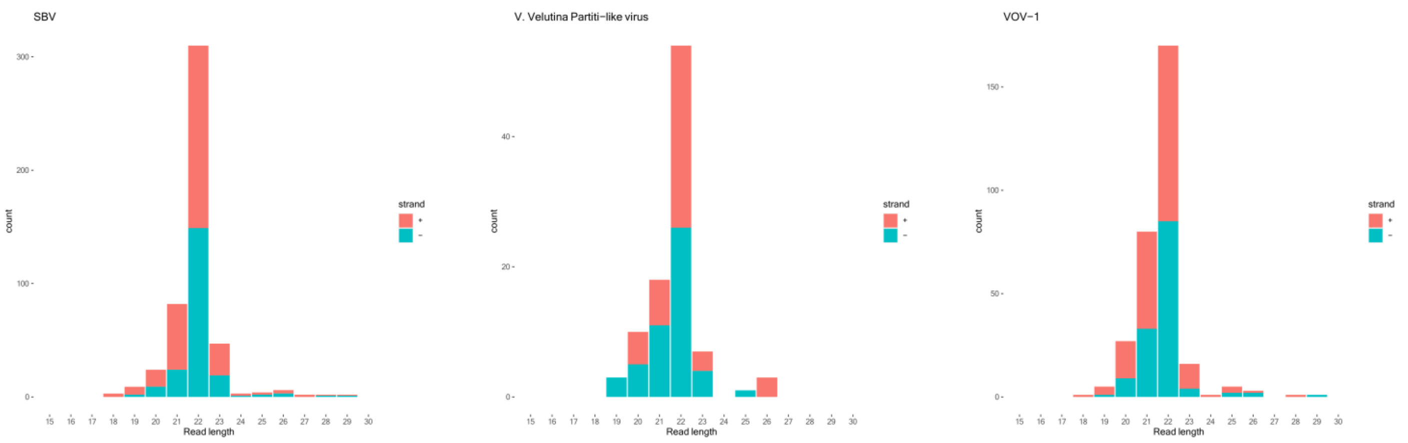

Supplementary Figure 3.4 : *Apis mellifera* – April – plant virus vsiRNA profiles

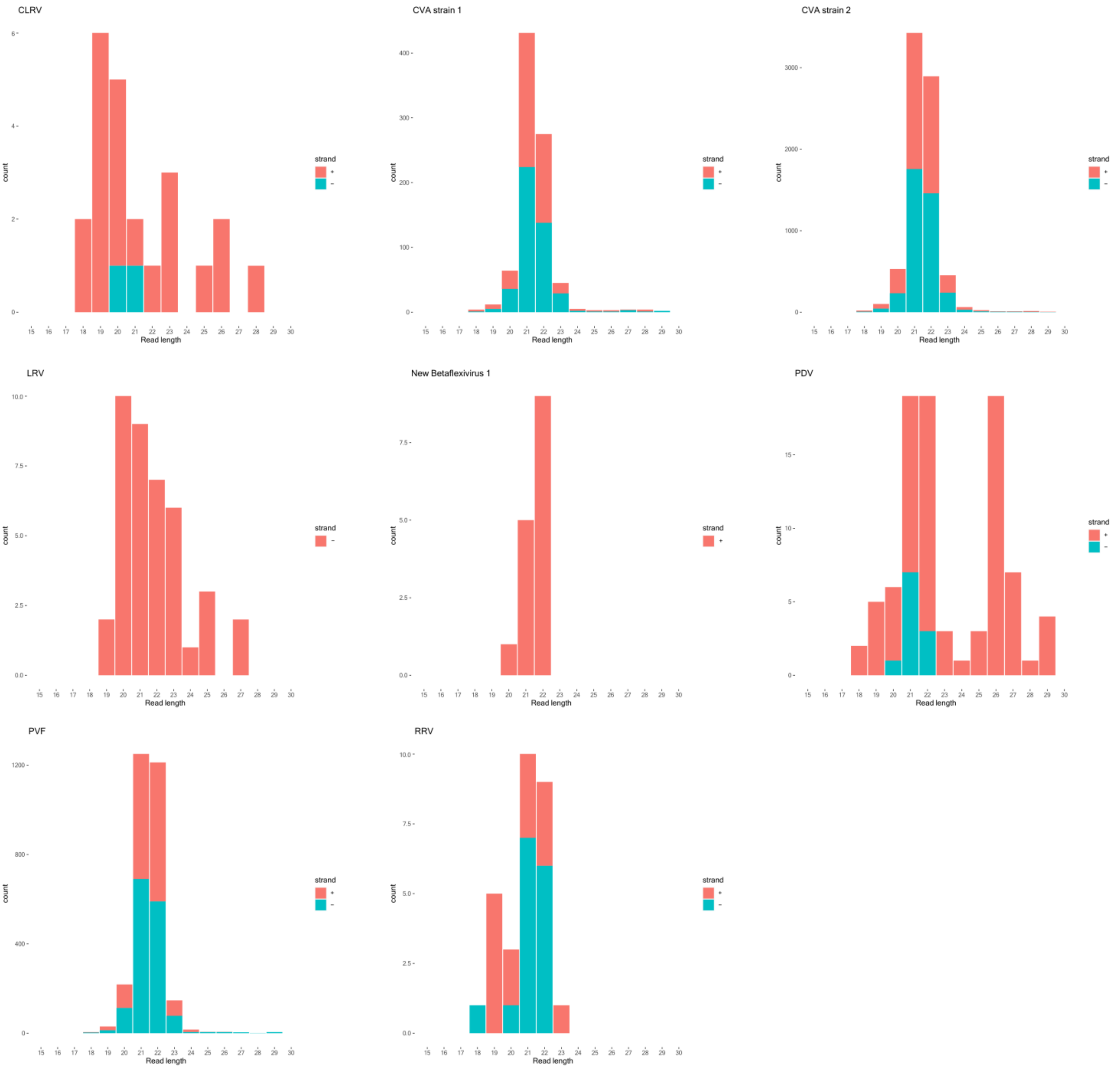

Supplementary Figure 3.5 : ***Apis mellifera*** **– June – insect virus vsiRNA profiles**

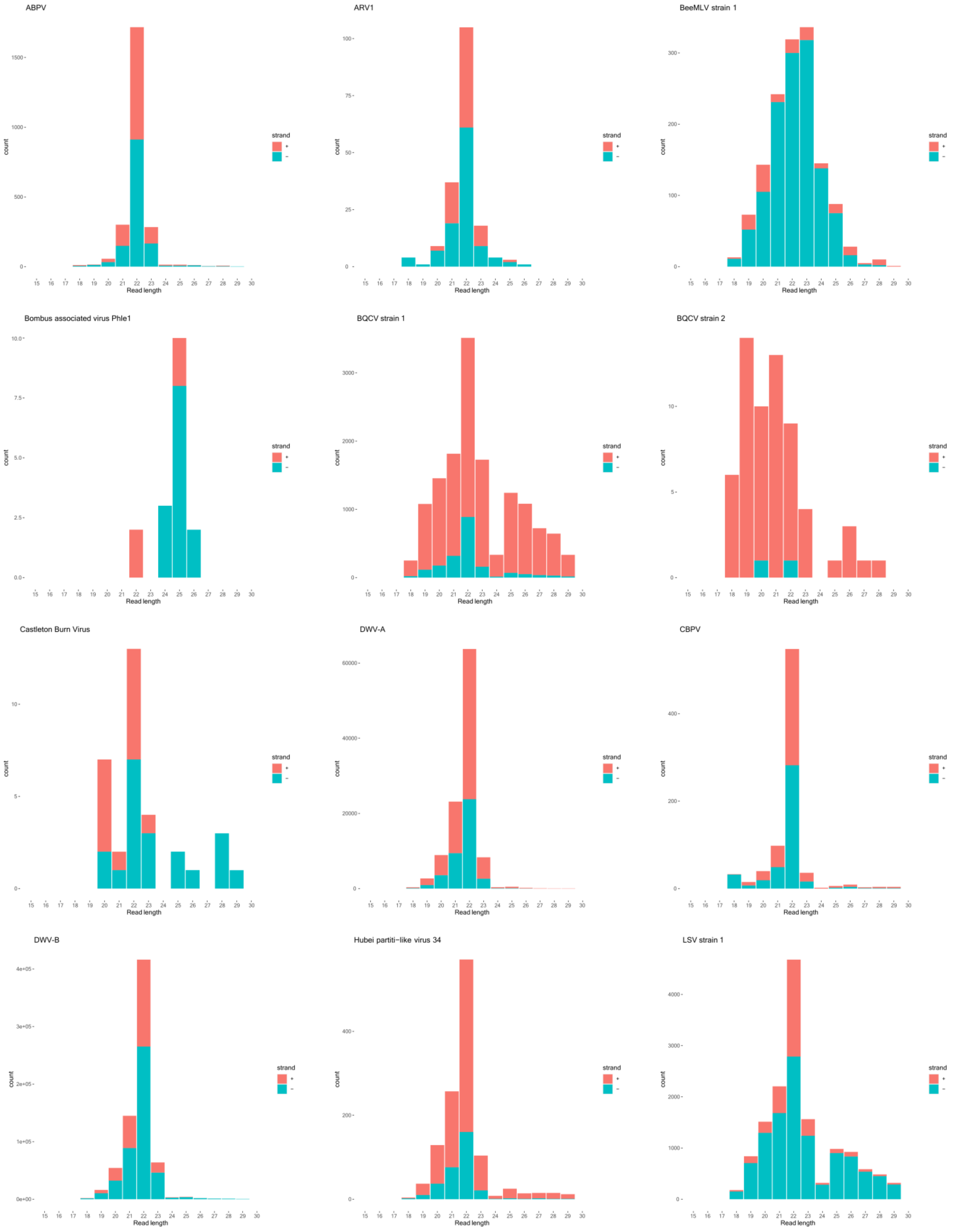

Supplementary Figure 3.6 : *Apis mellifera* – June – insect virus vsiRNA profiles

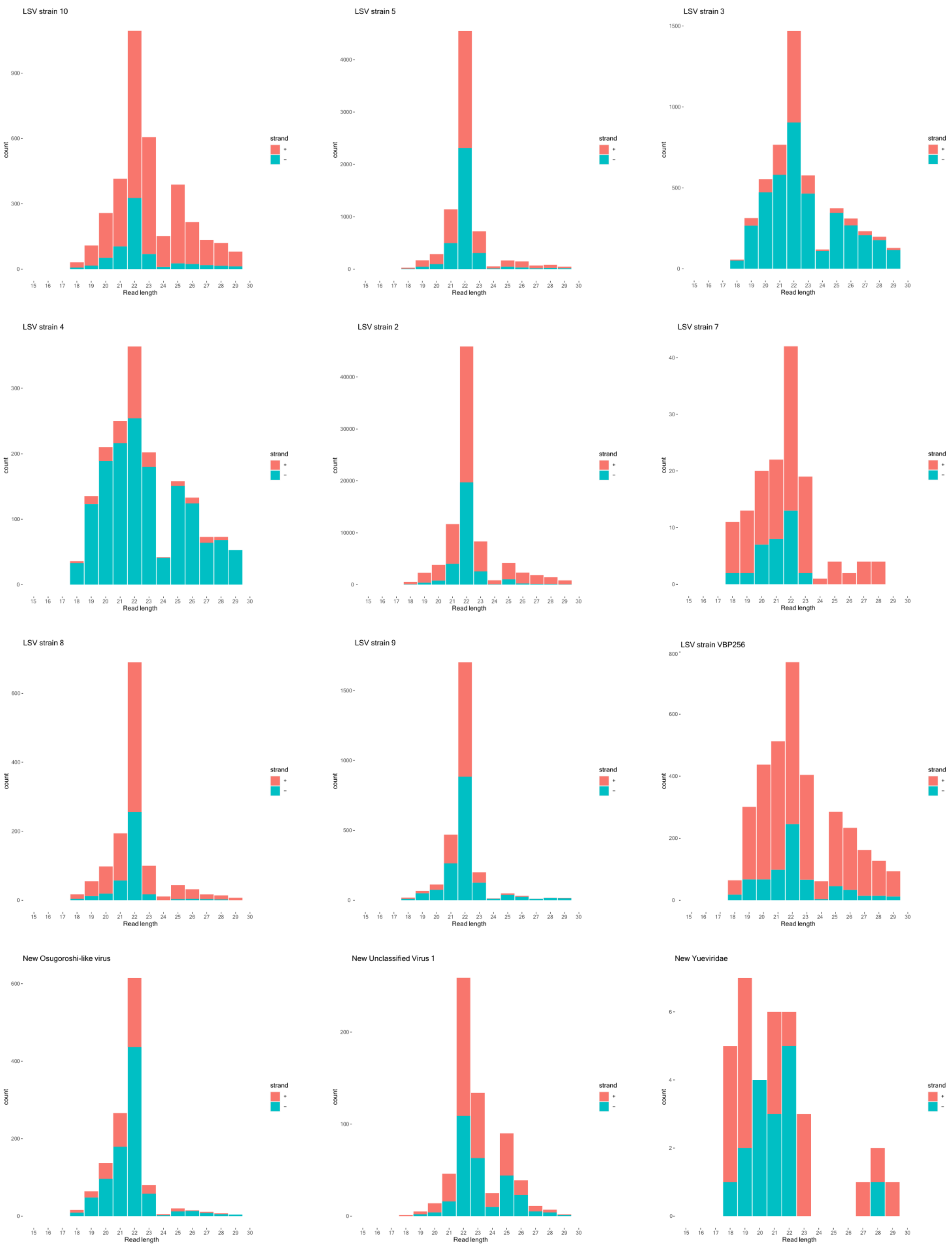

Supplementary Figure 3.7 : *Apis mellifera* – June – insect virus vsiRNA profiles

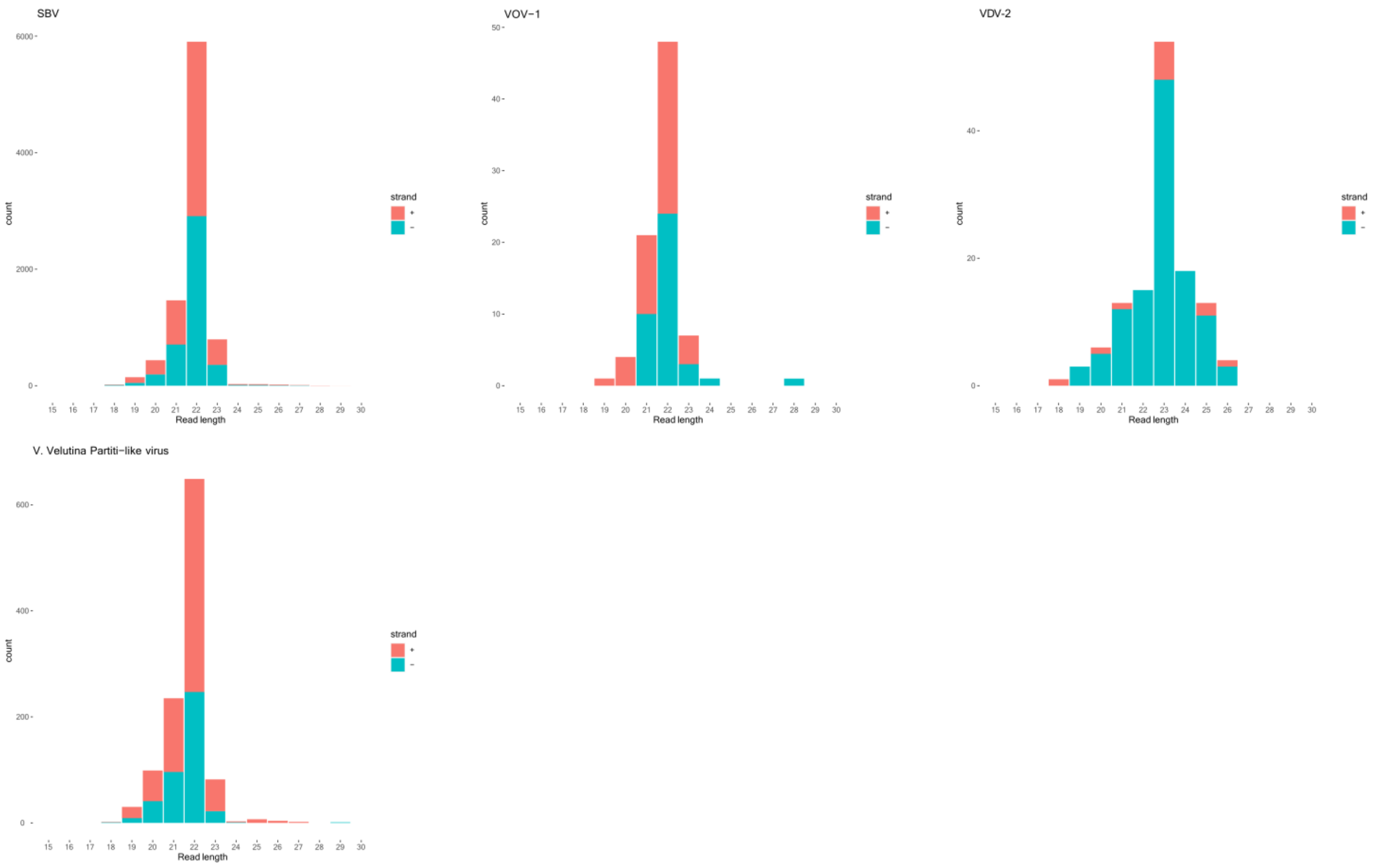

Supplementary Figure 3.8 : *Apis mellifera* – June – plant virus vsiRNA profiles

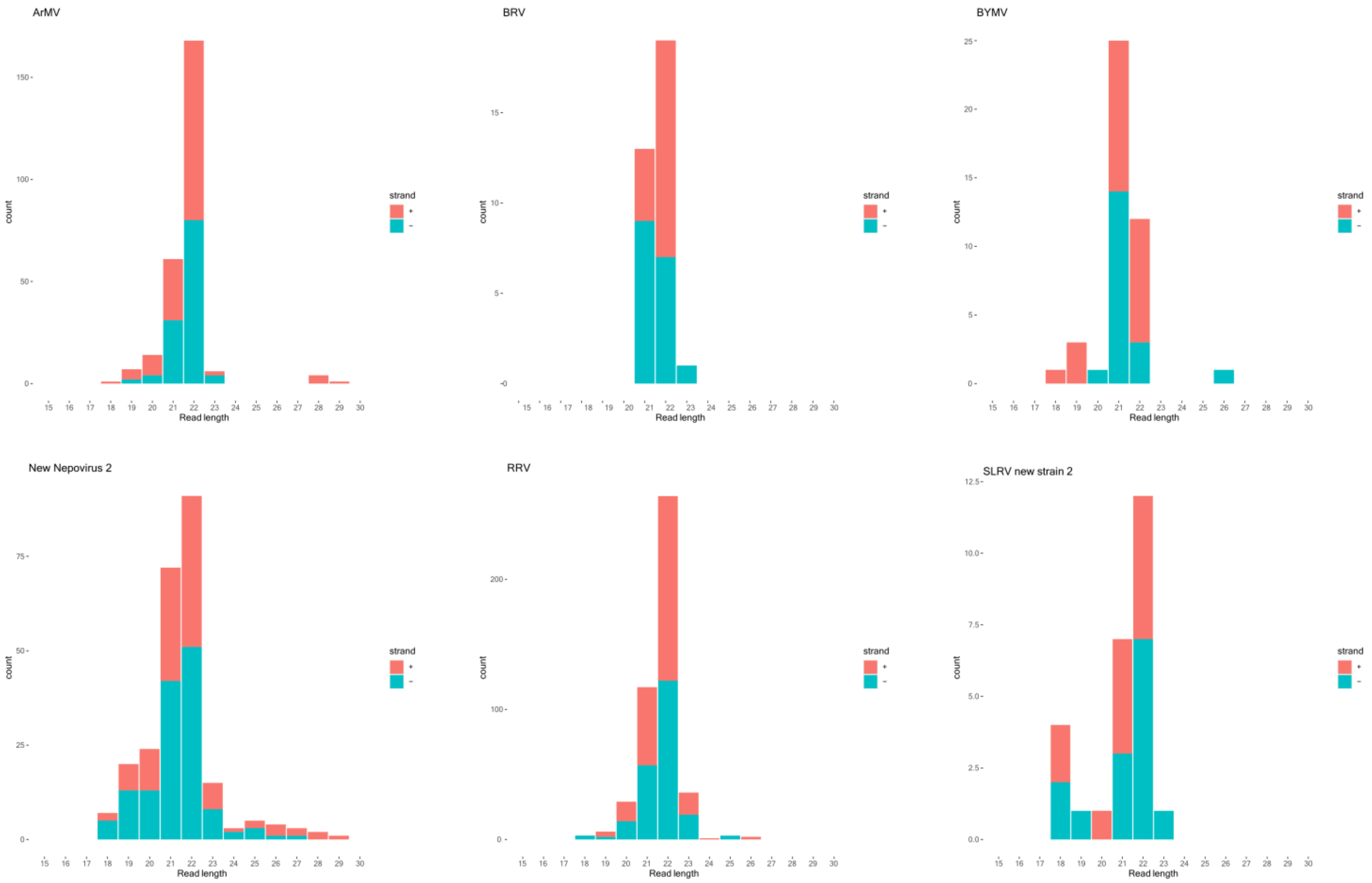

Supplementary Figure 3.9 : *Apis mellifera* – June – plant virus vsiRNA profiles

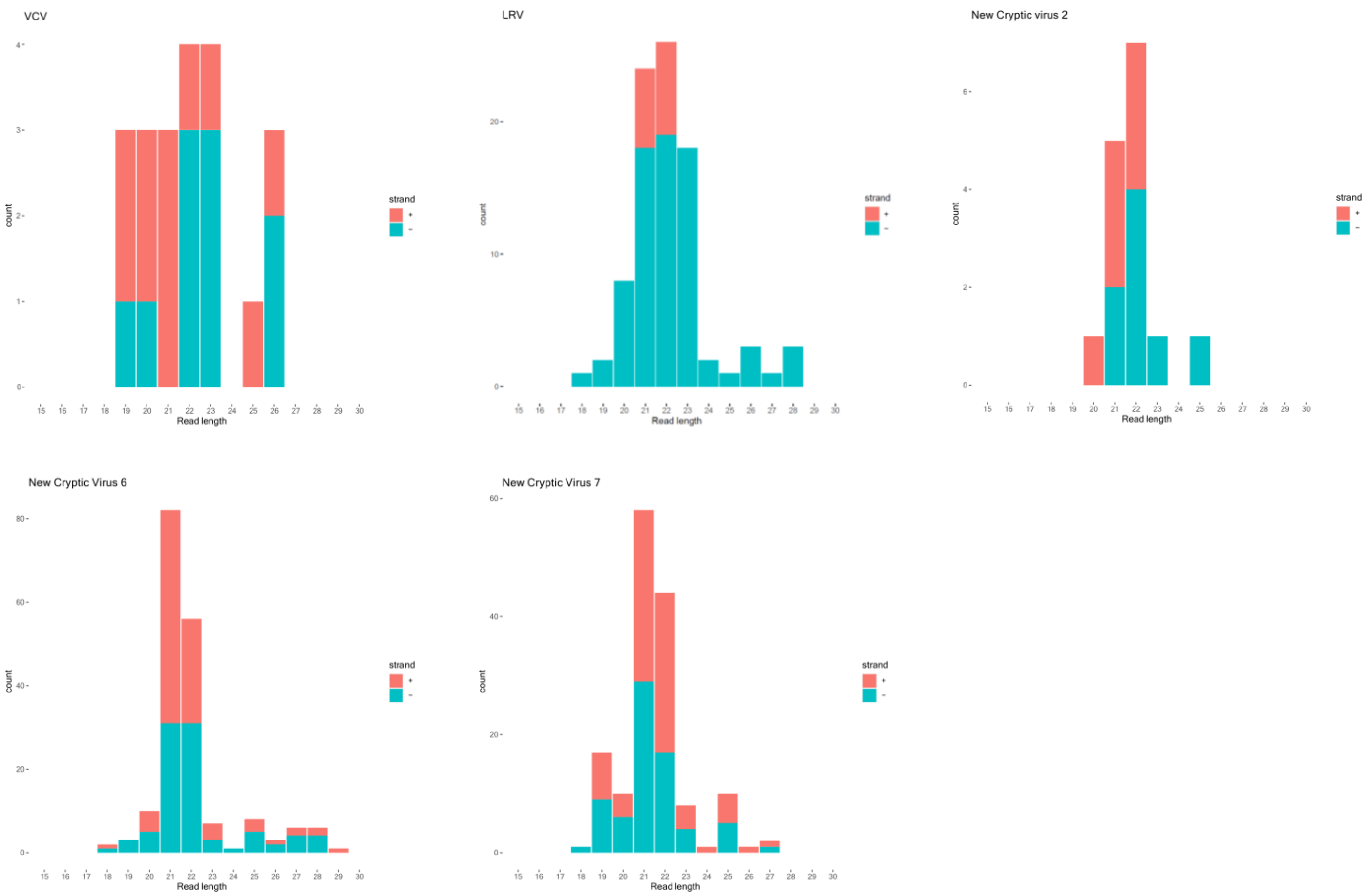

Supplementary Figure 3.10 : *Apis mellifera* – August – insect virus vsiRNA profiles

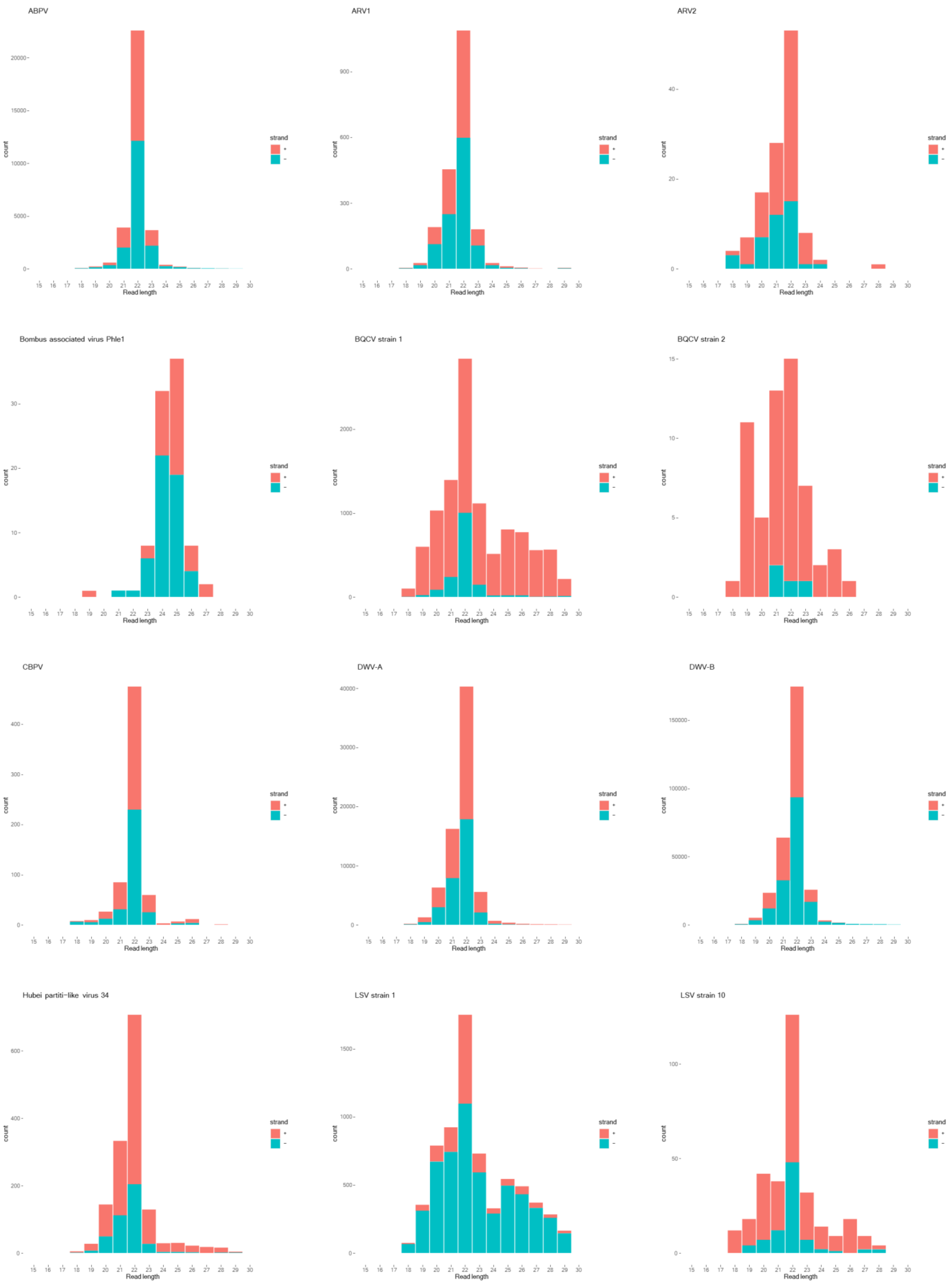

Supplementary Figure 3.11 : *Apis mellifera* – August – insect virus vsiRNA profiles

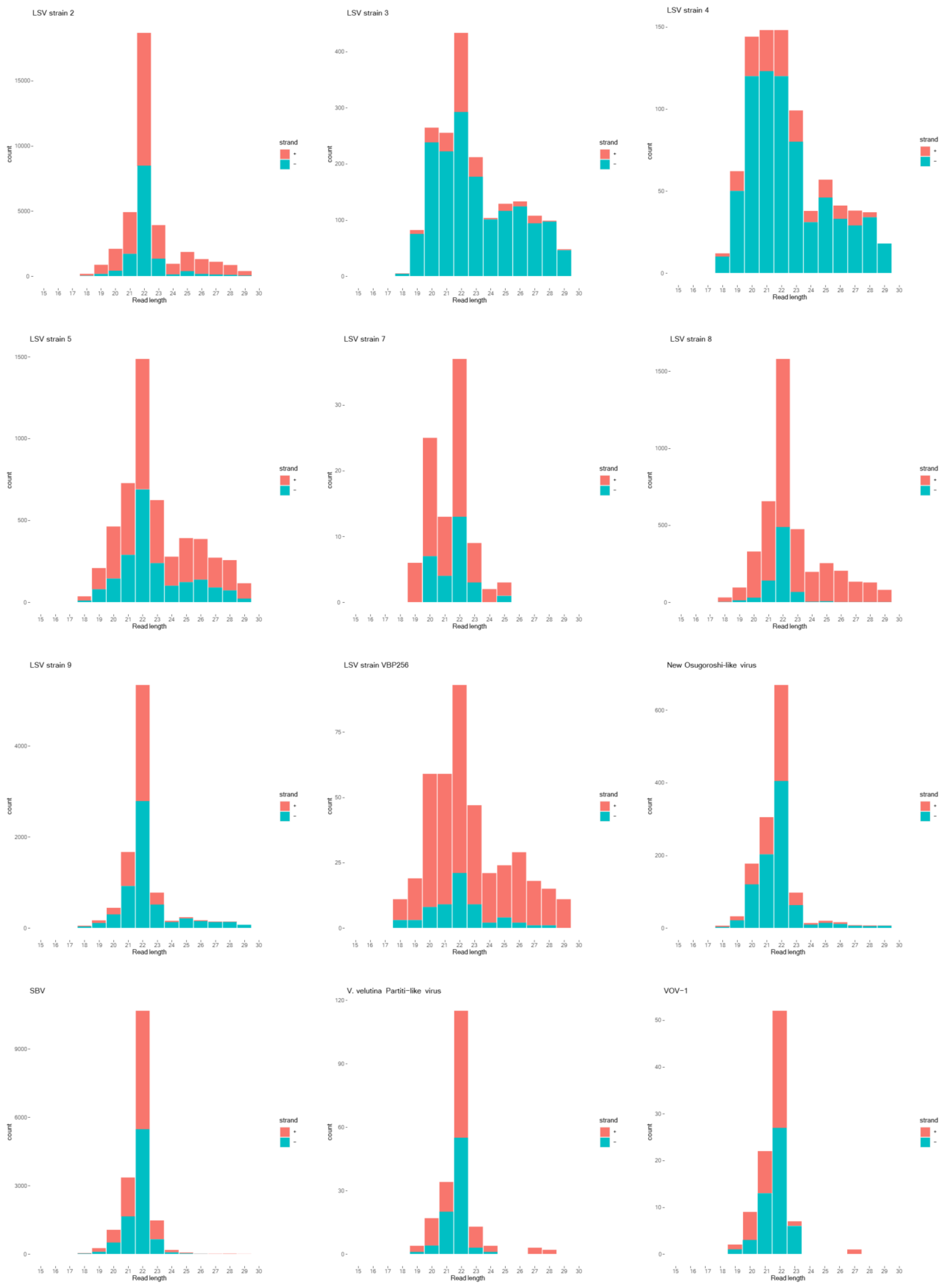

Supplementary Figure 3.12 : *Apis mellifera* – August – plant virus vsiRNA profiles

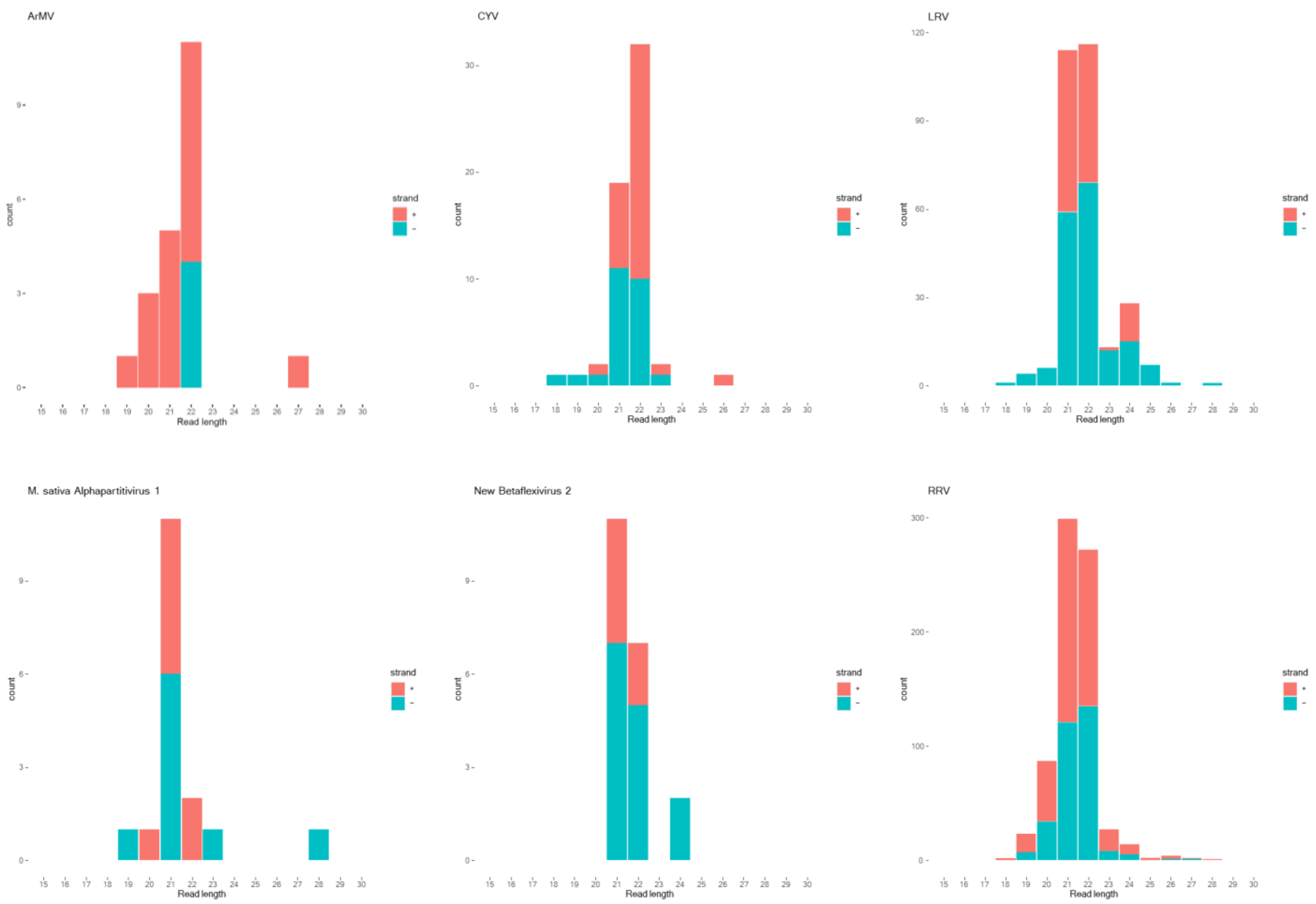

Supplementary Figure 3.13 : *Bombus terrestris* – April – insect virus vsiRNA profiles

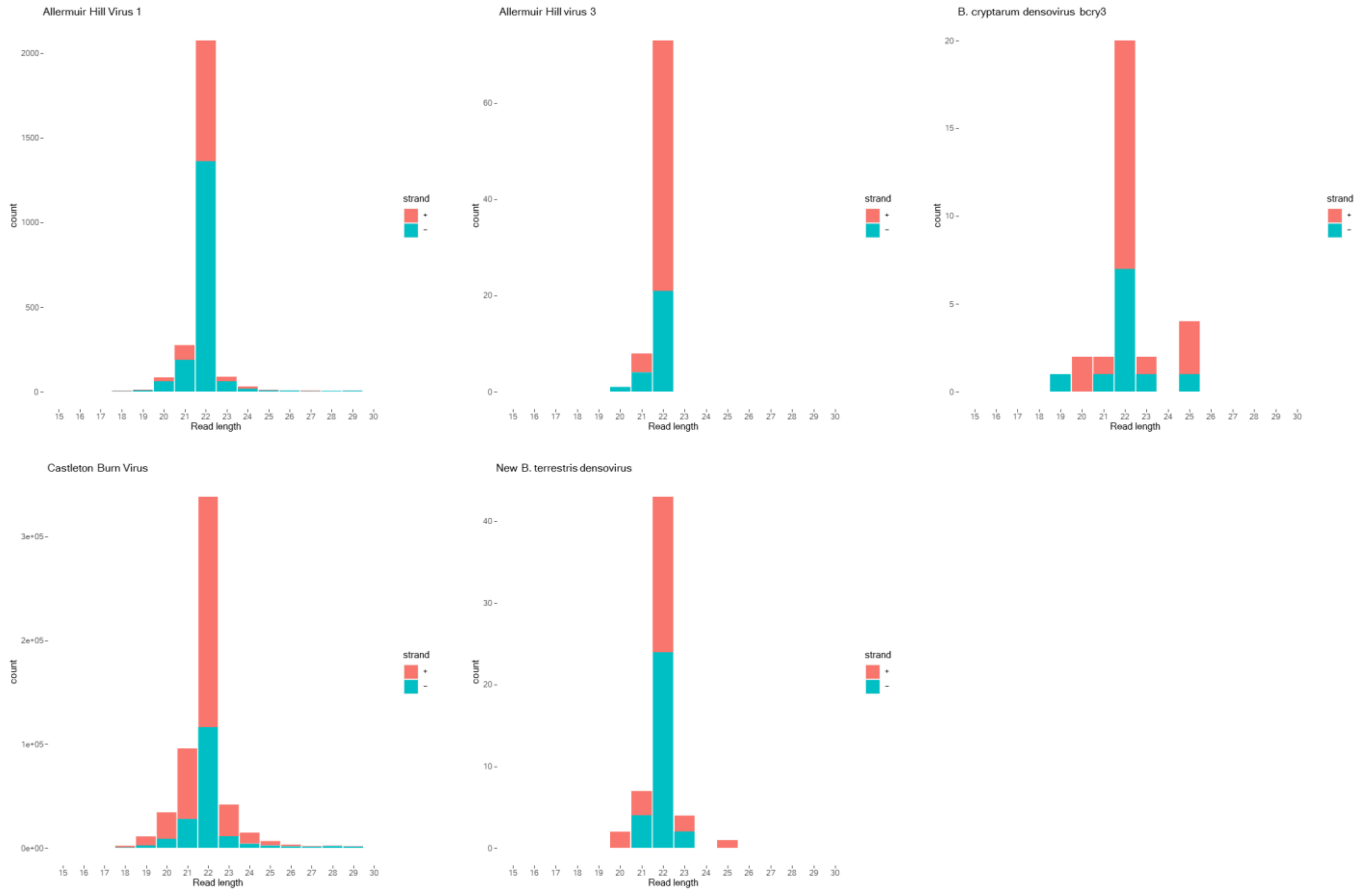

Supplementary Figure 3.14 : *Bombus terrestris* – April – plant virus vsiRNA profiles

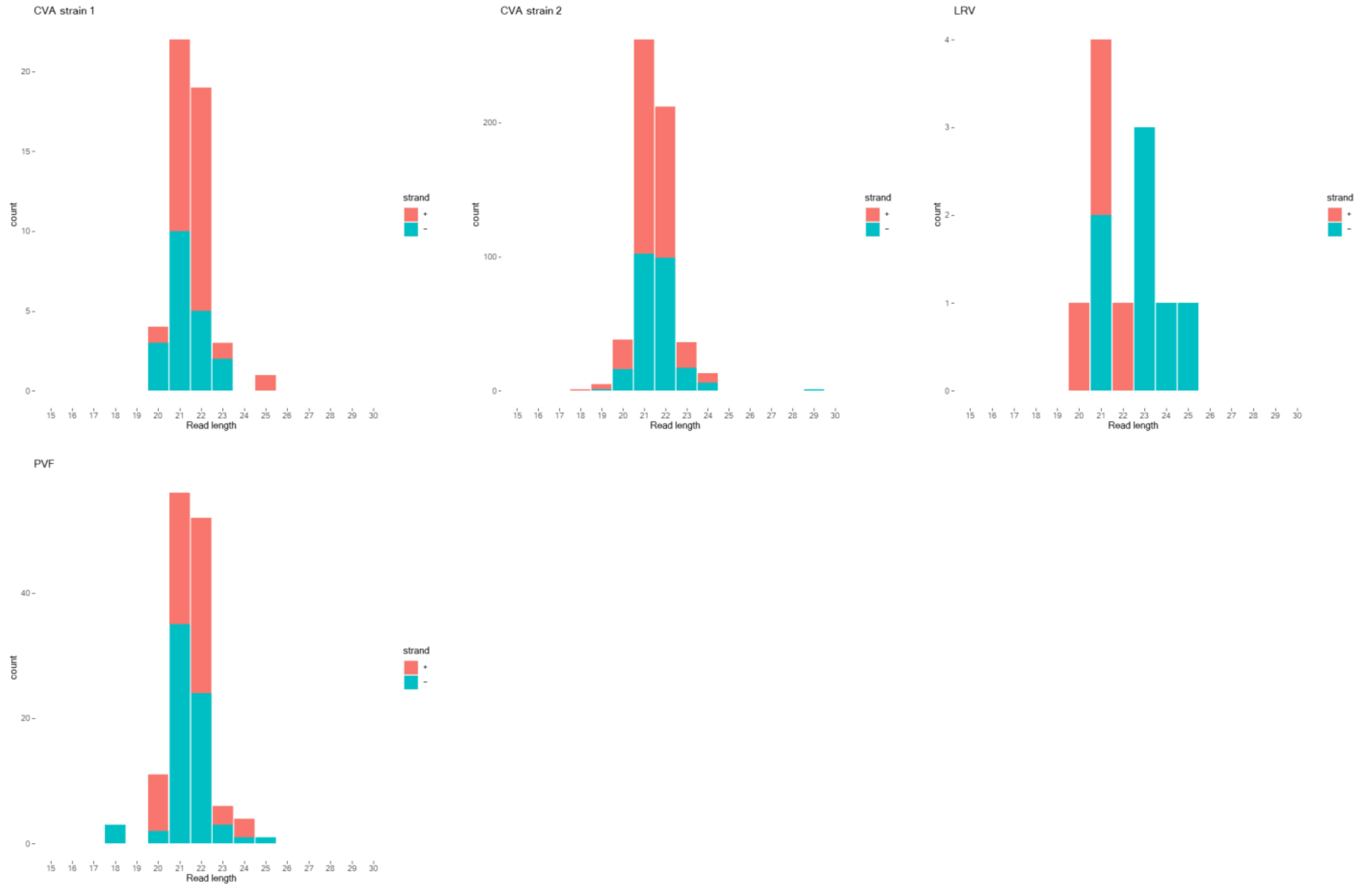

Supplementary Figure 3.15 : *Bombus terrestris* – June – insect virus vsiRNA profiles

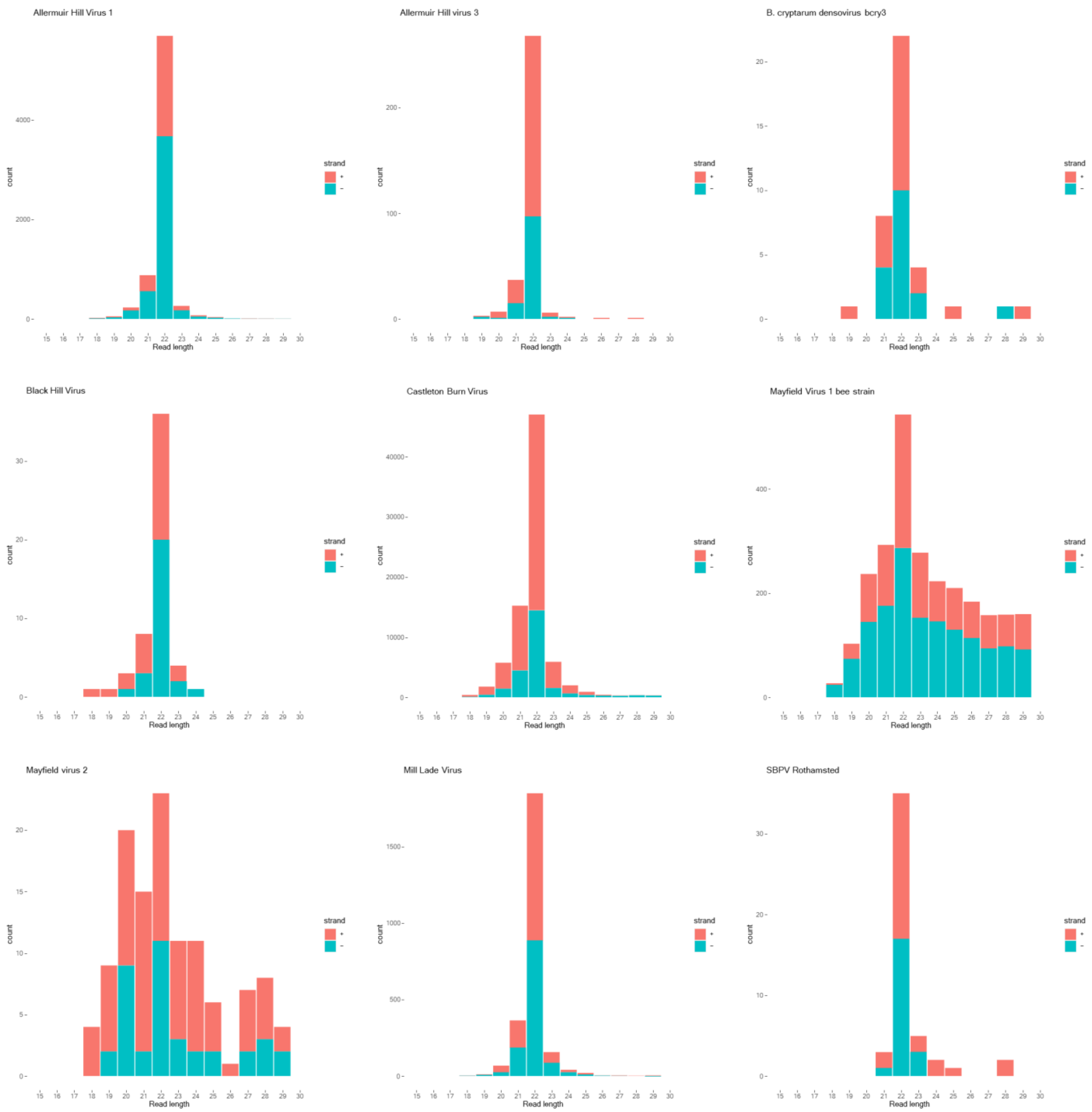

Supplementary Figure 3.16 : *Bombus terrestris* – June – plant virus vsiRNA profiles

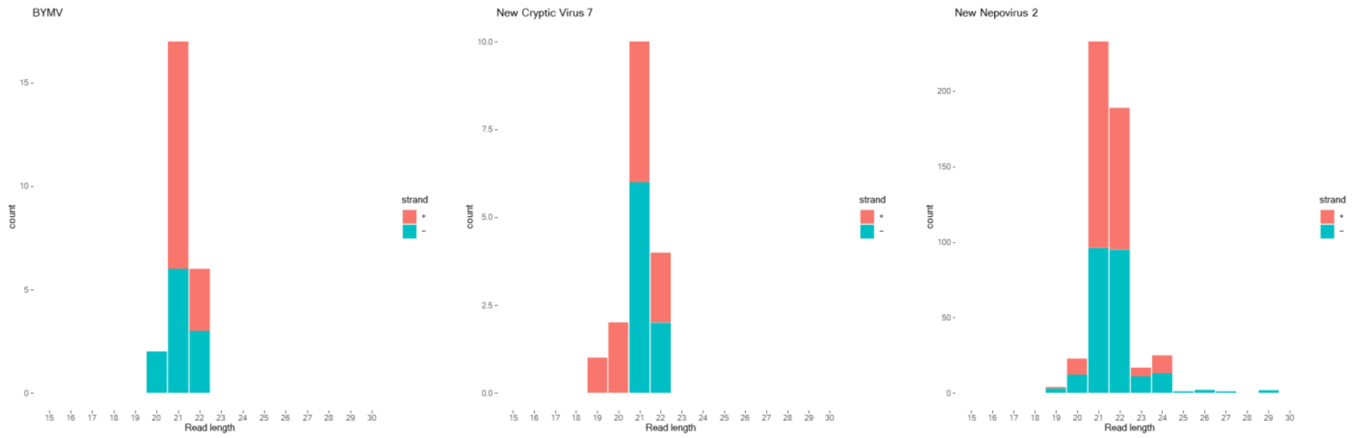

Supplementary Figure 3.17 : *Bombus terrestris* – August – insect virus vsiRNA profiles

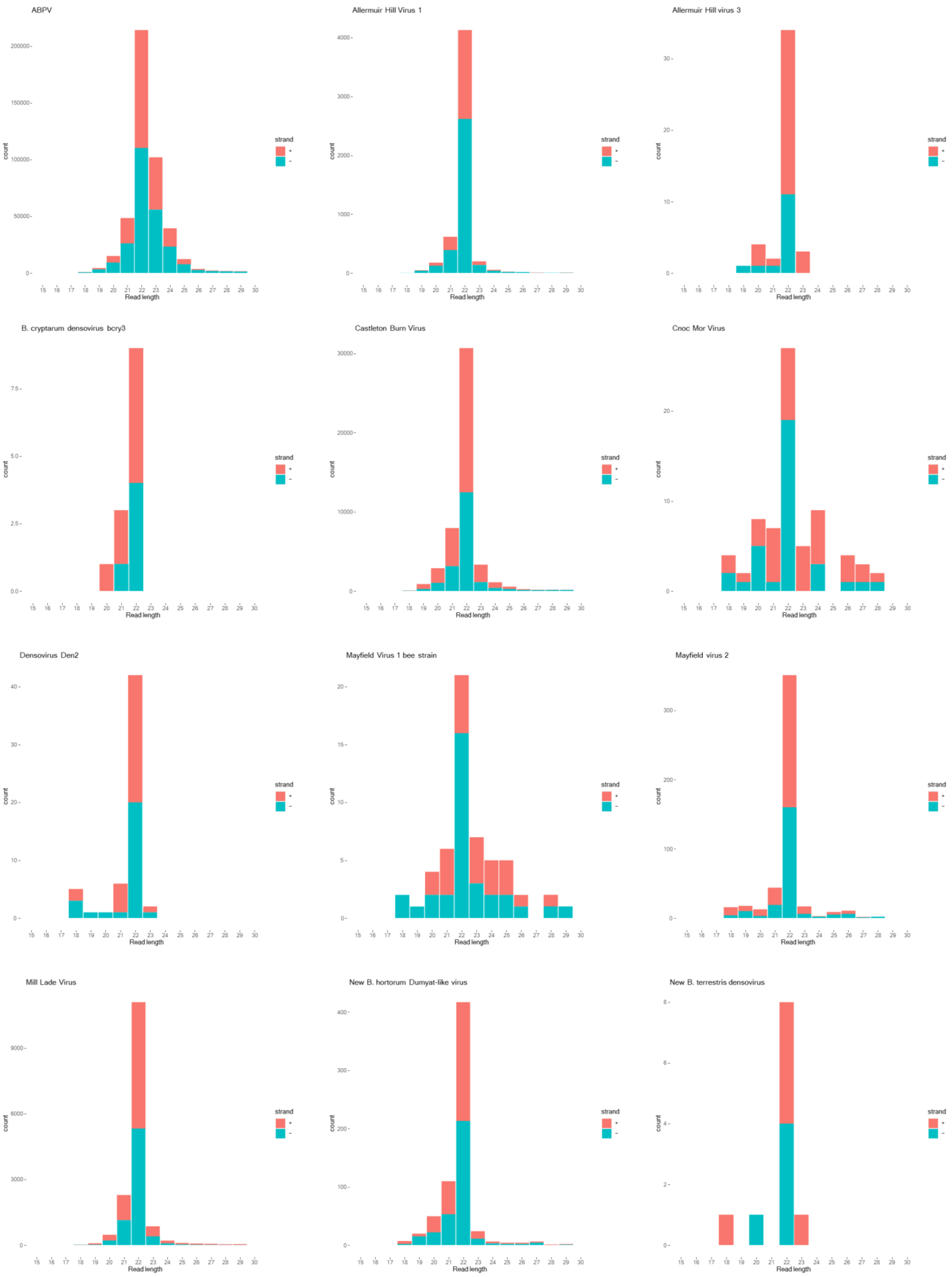

Supplementary Figure 3.18 : *Bombus terrestris* – August – insect virus vsiRNA profiles

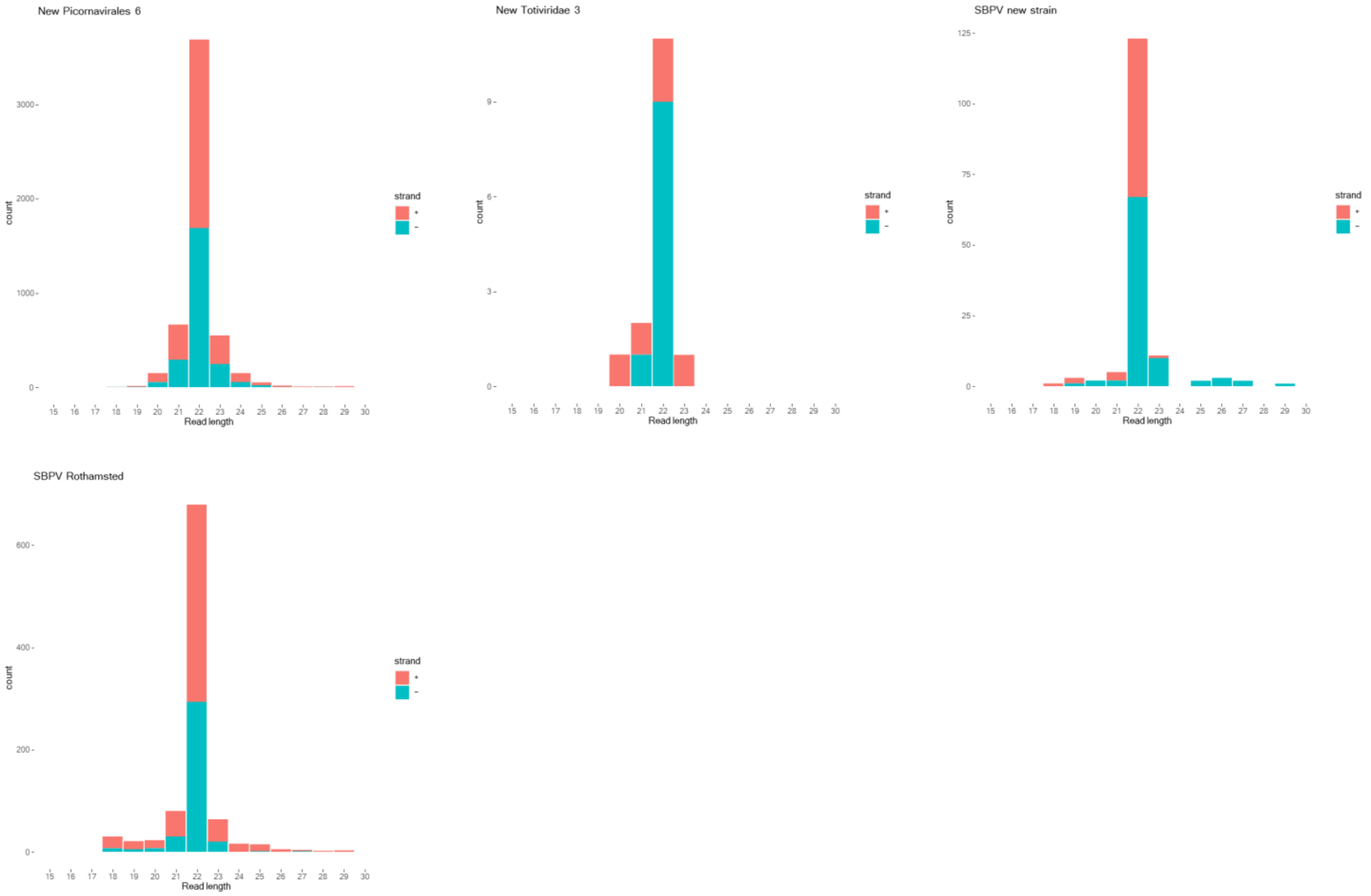

Supplementary Figure 3.19 : *Bombus terrestris* – August – plant virus vsiRNA profiles

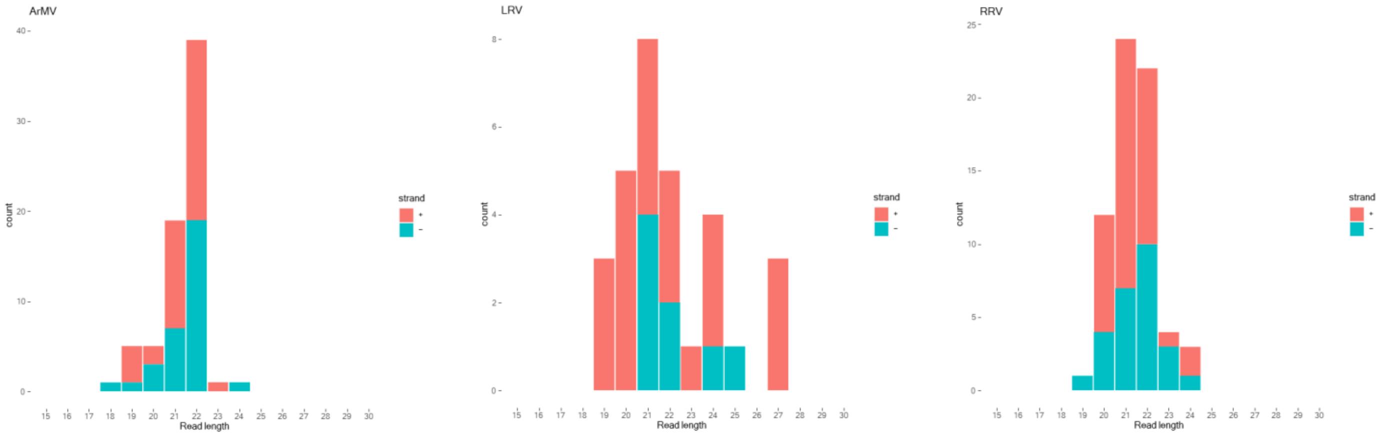

Supplementary Figure 3.20 : *Bombus lapidarius* – April – insect virus vsiRNA profiles

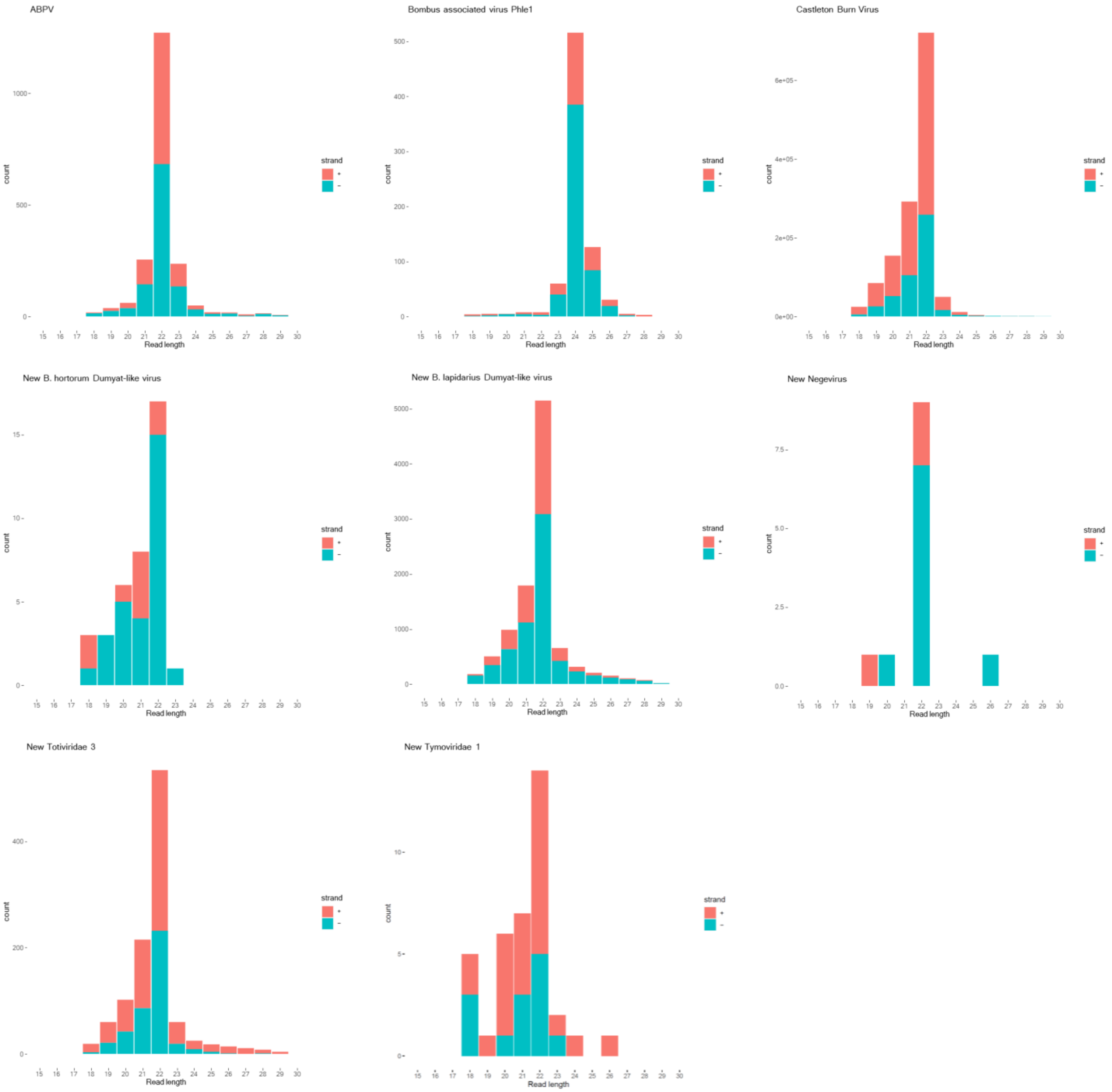

Supplementary Figure 3.21 : *Bombus lapidarius* – April – plant virus vsiRNA profiles

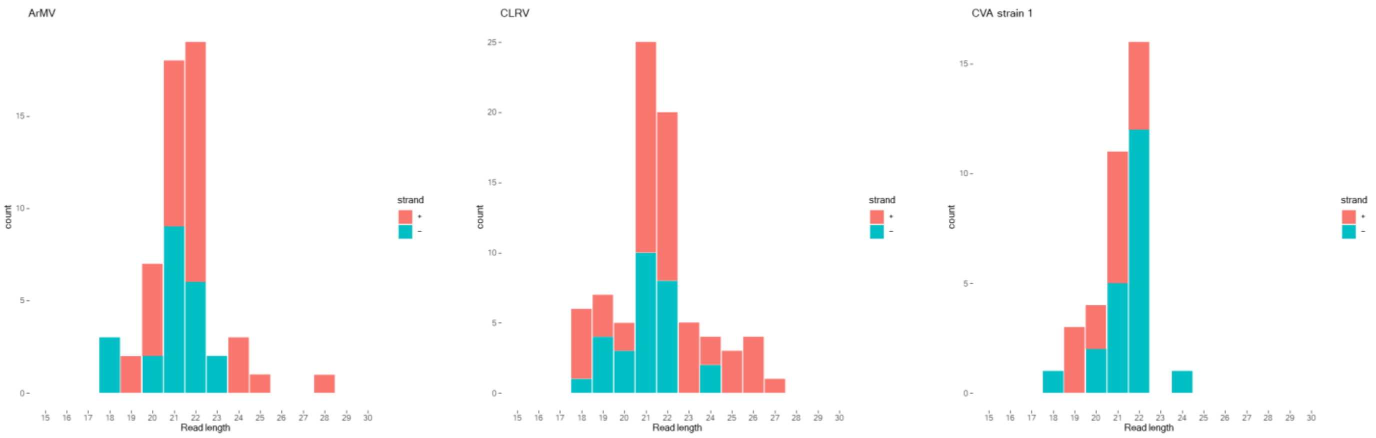

Supplementary Figure 3.22 : *Bombus lapidarius* – April – plant virus vsiRNA profiles

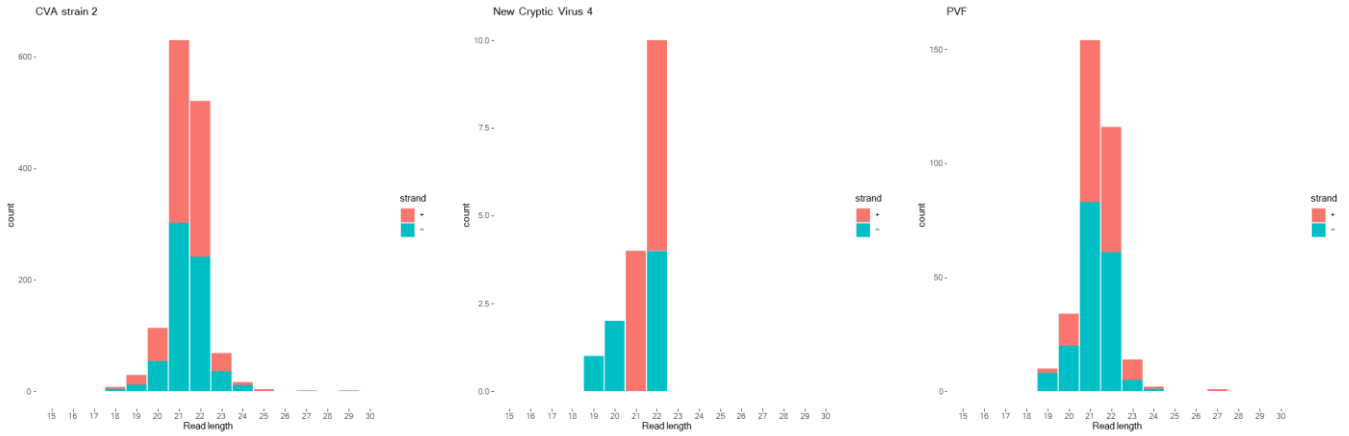

Supplementary Figure 3.23 : *Bombus lapidarius* – June – insect virus vsiRNA profiles

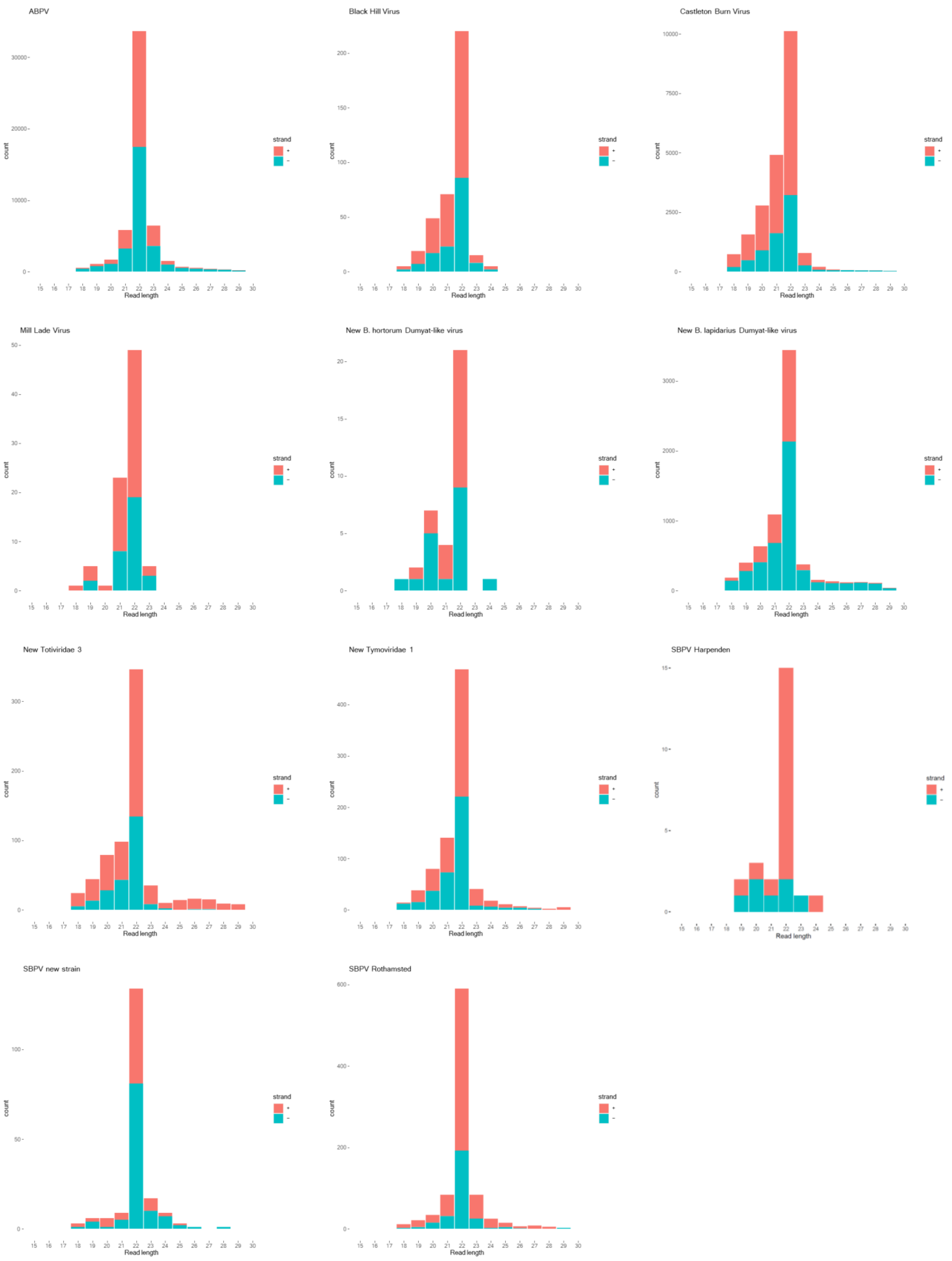

Supplementary Figure 3.24 : *Bombus lapidarius* – June – plant virus vsiRNA profiles

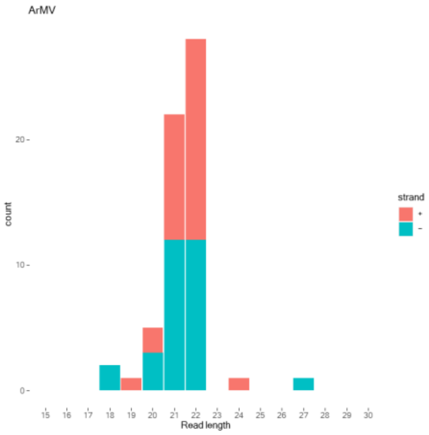

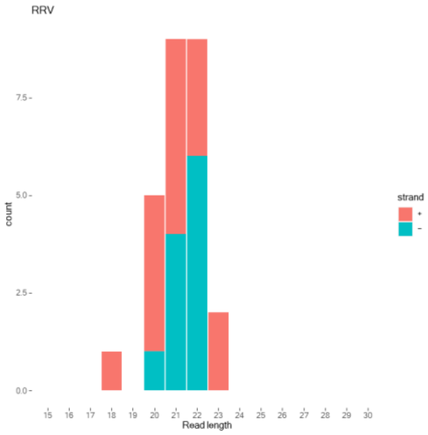

Supplementary Figure 3.25 : *Bombus lapidarius* – August – insect virus vsiRNA profiles

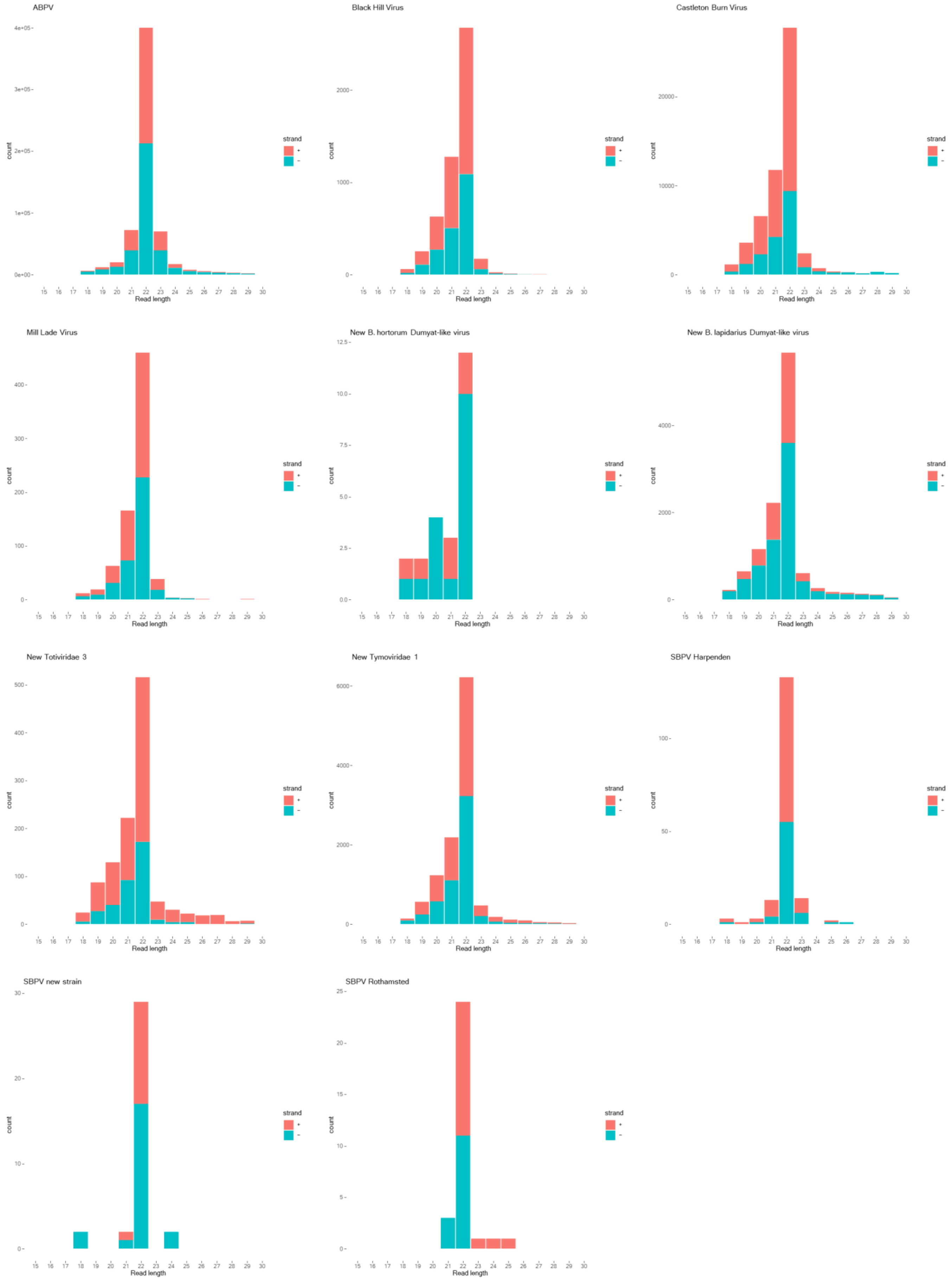

Supplementary Figure 3.26 : *Bombus lapidarius* – August – plant virus vsiRNA profiles

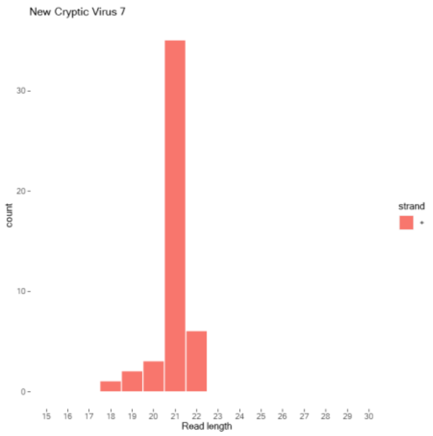

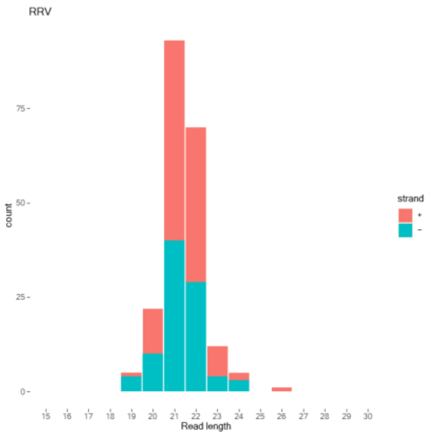

Supplementary Figure 3.27 : *Bombus hortorum* – June – insect virus vsiRNA profiles

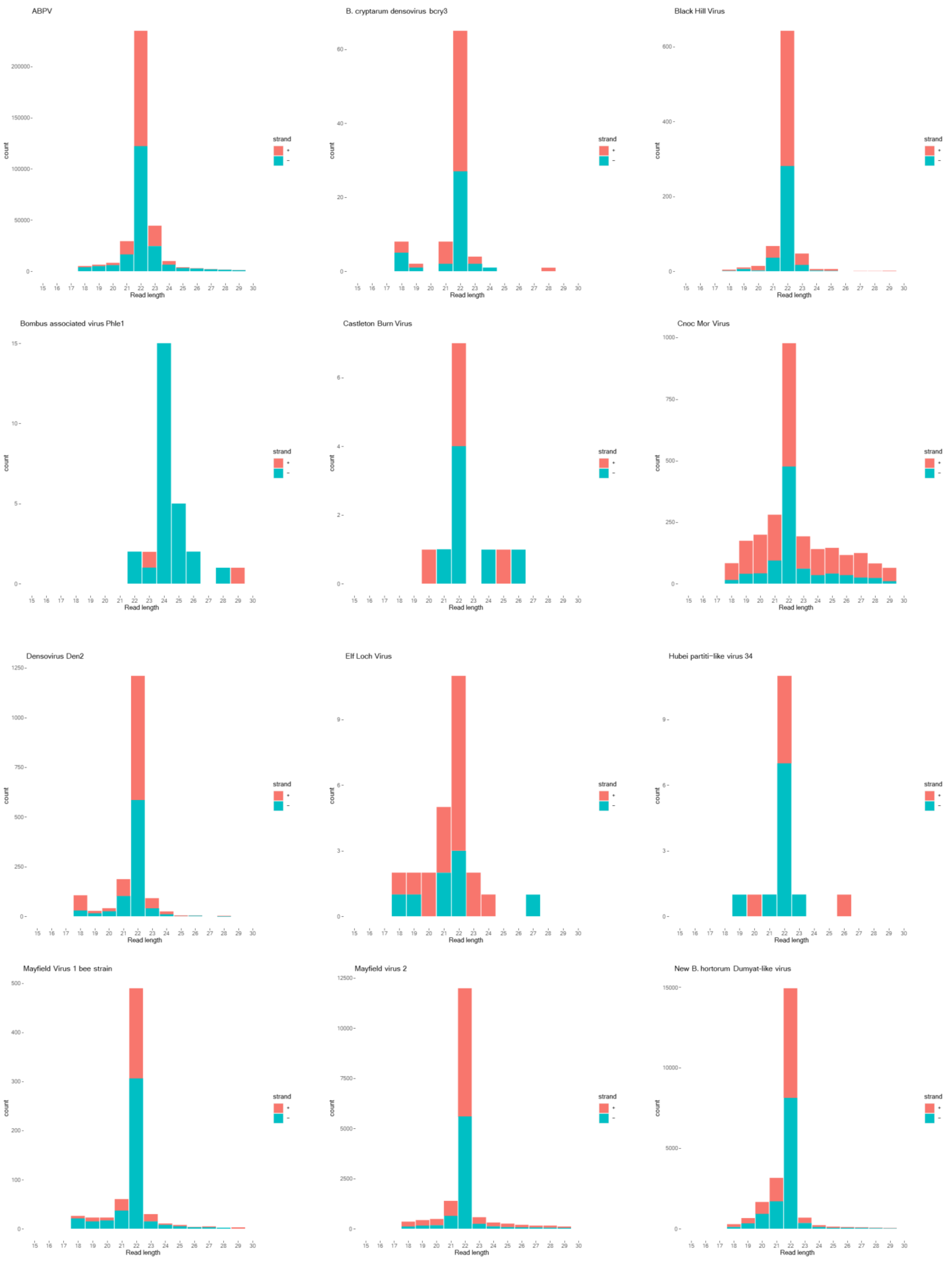

Supplementary Figure 3.28 : *Bombus hortorum* – June – insect virus vsiRNA profiles

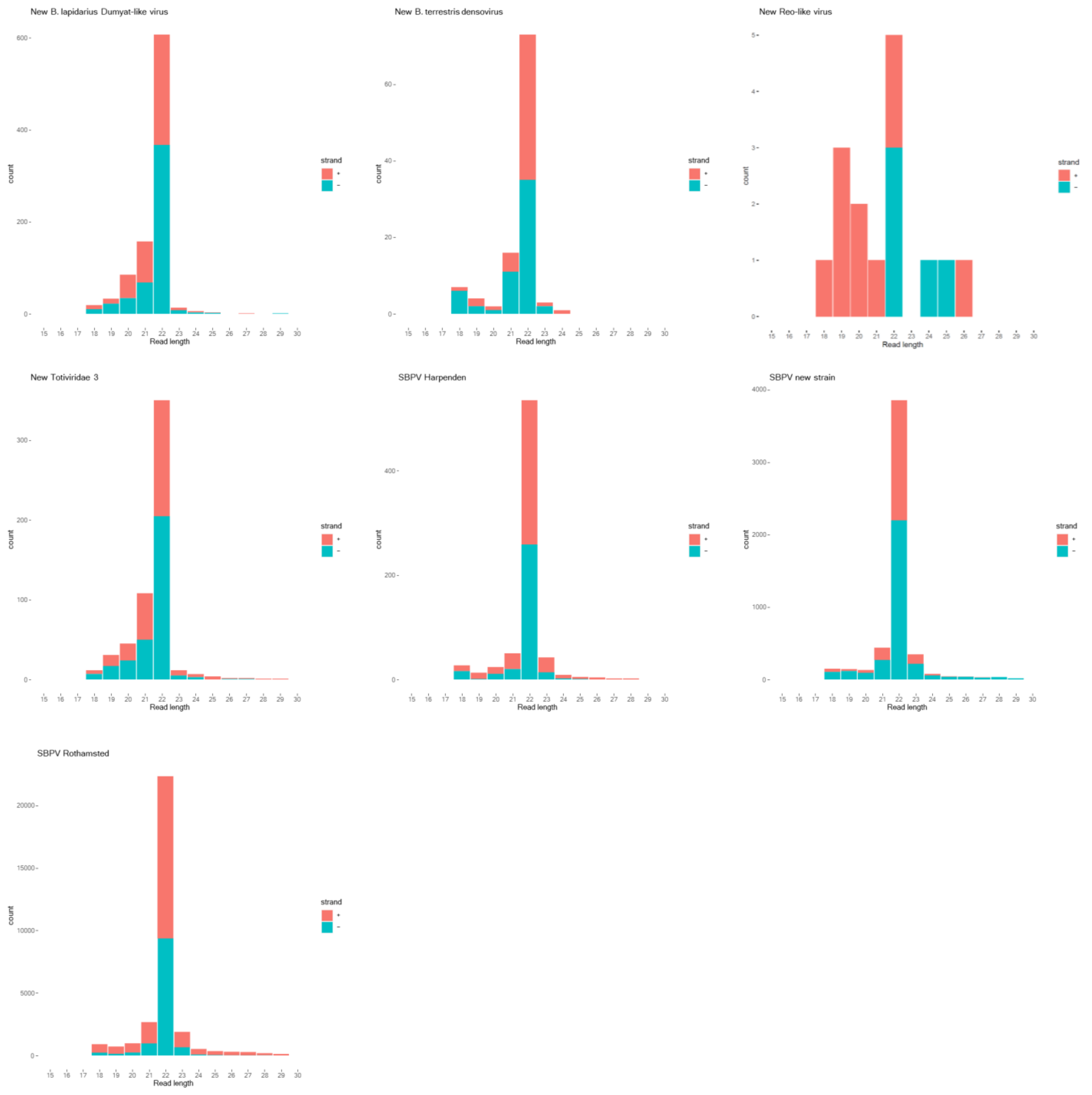

Supplementary Figure 3.29 : *Bombus hortorum* – June – plant virus vsiRNA profiles

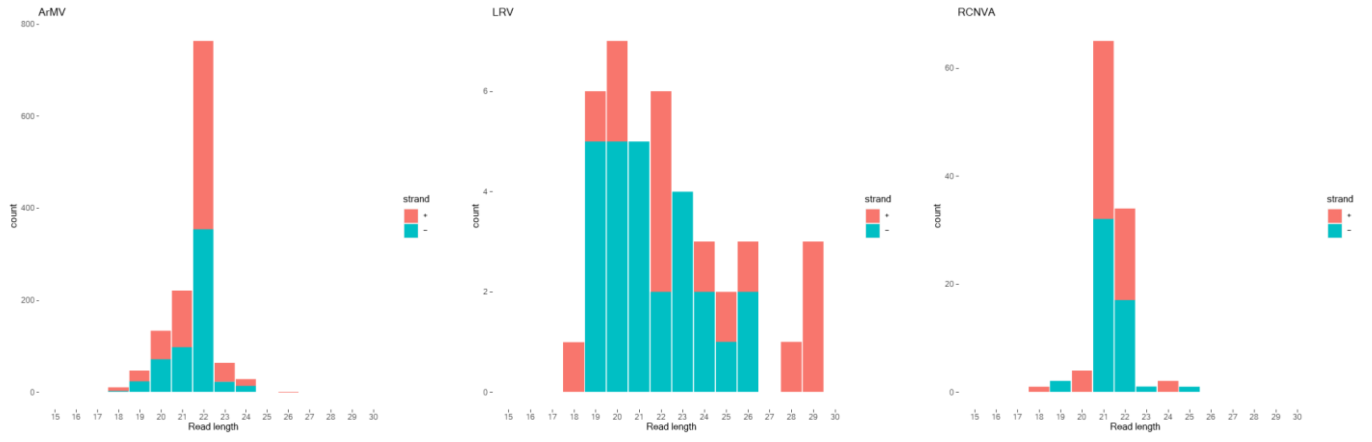

Supplementary Figure 3.30 : *Bombus hortorum* – June – plant virus vsiRNA profile

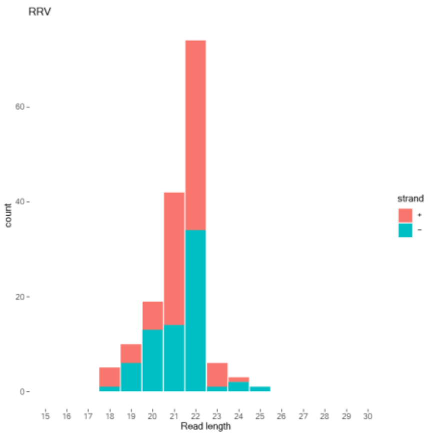

Supplementary Figure 3.31 : *Bombus hortorum* – August – insect virus vsiRNA profiles

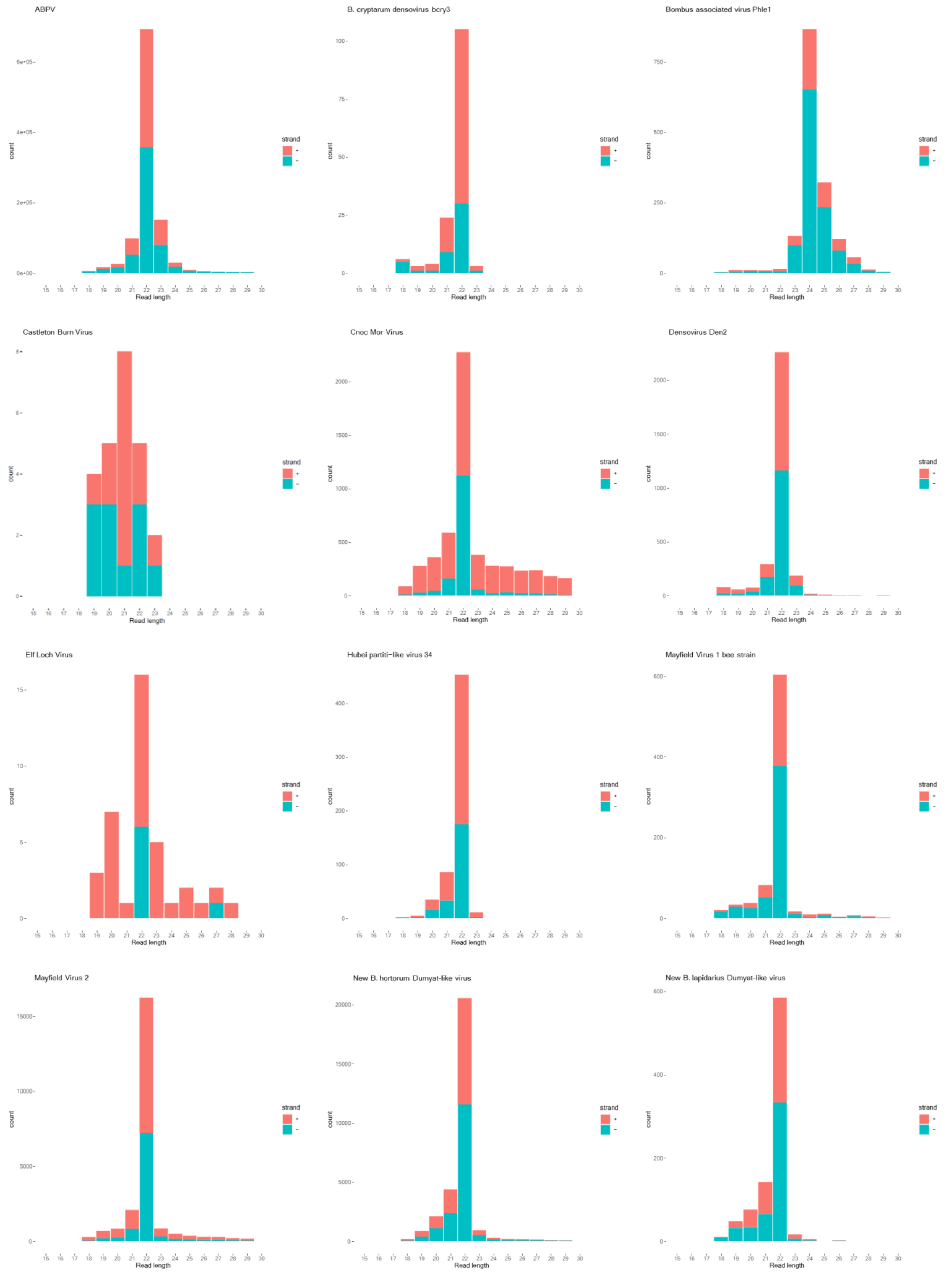

Supplementary Figure 3.32 : *Bombus hortorum* – August – insect virus vsiRNA profiles

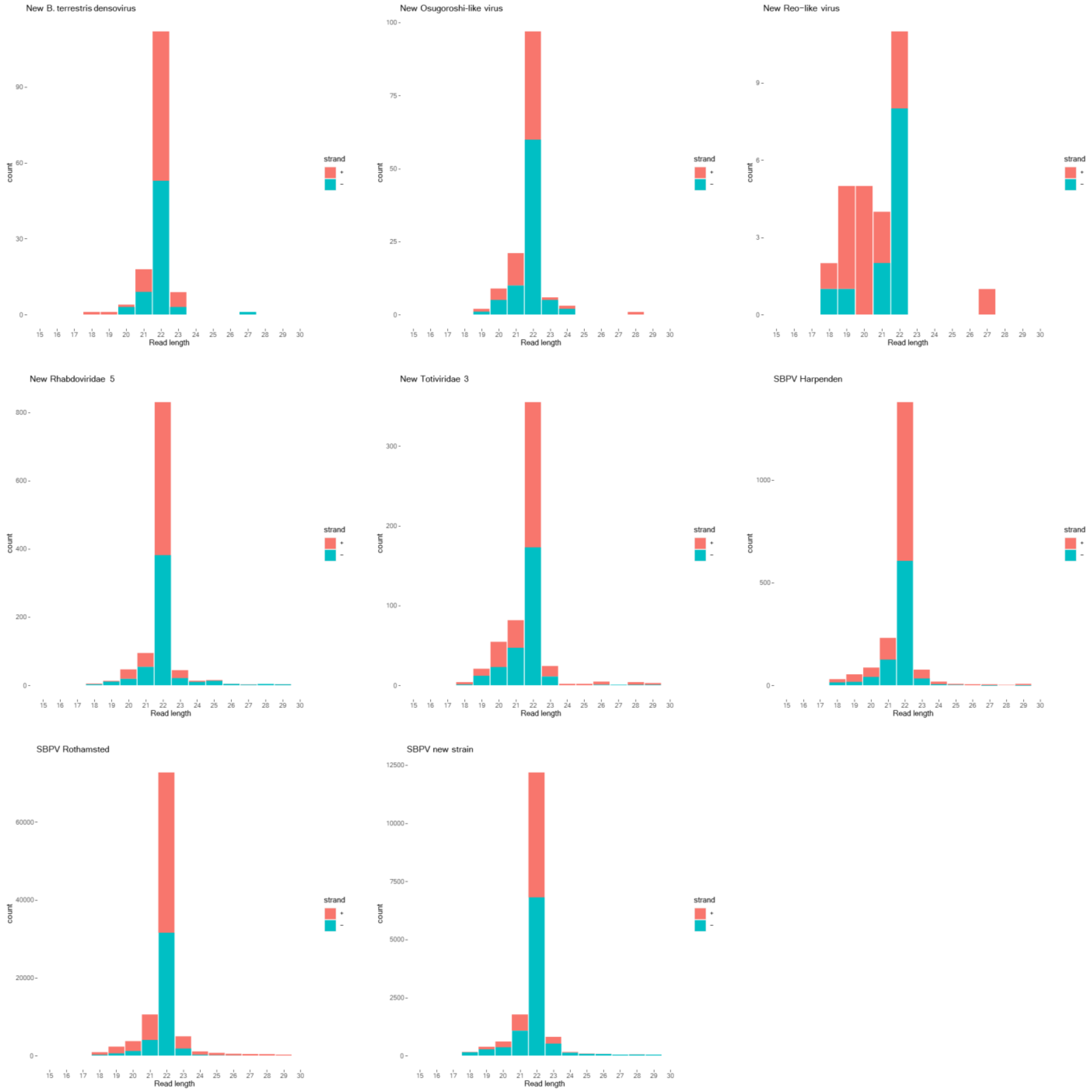

Supplementary Figure 3.33 : *Bombus hortorum* – August – plant virus vsiRNA profiles

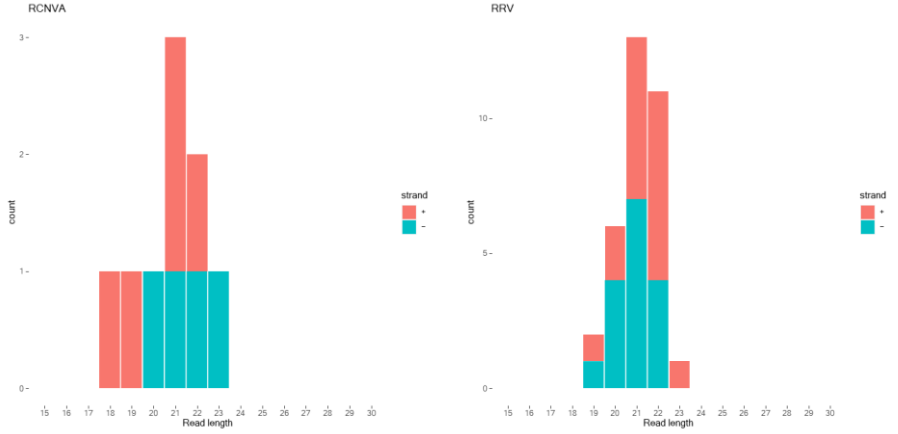

Supplementary Figure 3.34 : *Bombus pascuorum* – June – insect virus vsiRNA profiles

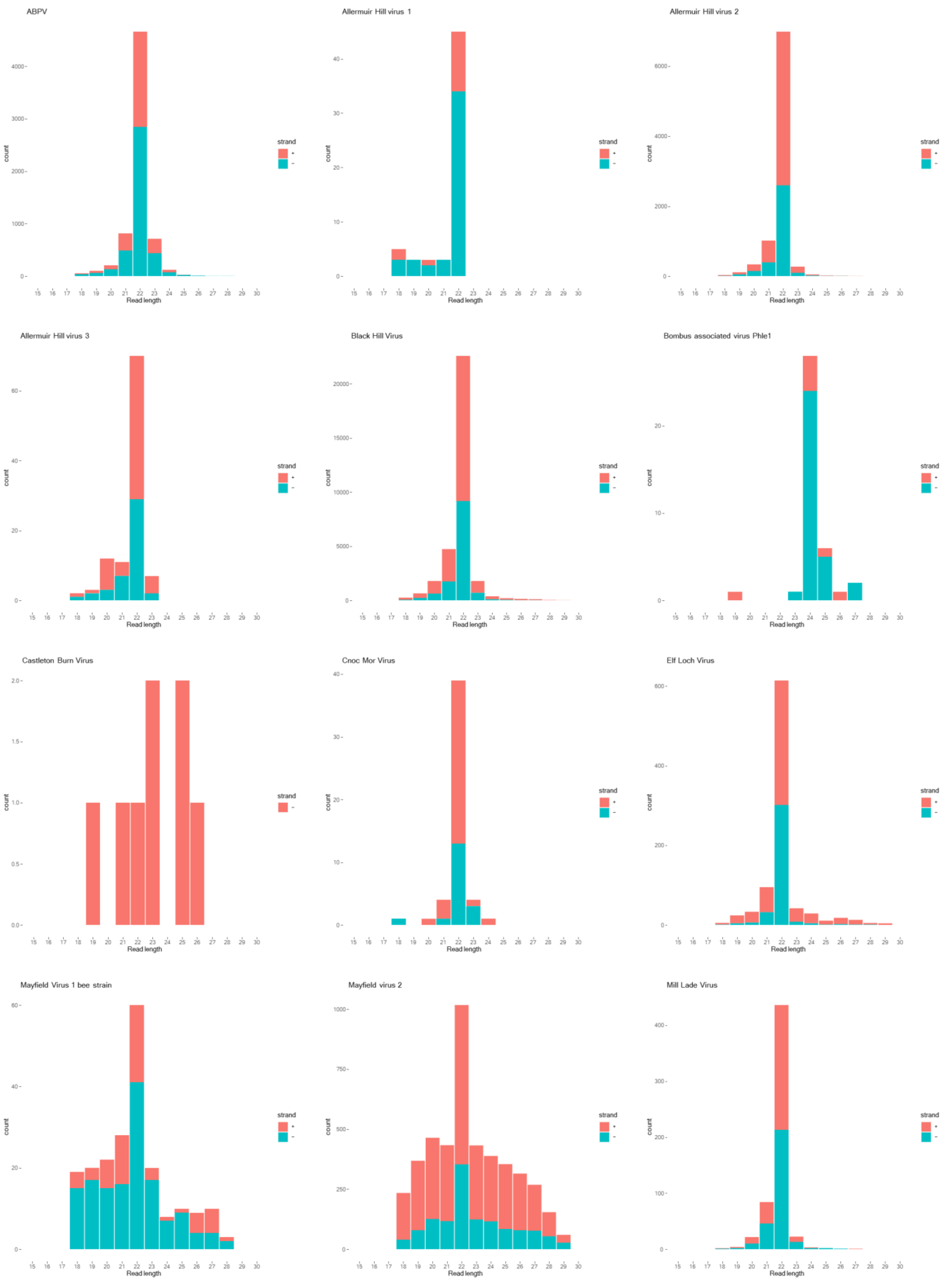

Supplementary Figure 3.35 : *Bombus pascuorum* – June – insect virus vsiRNA profiles

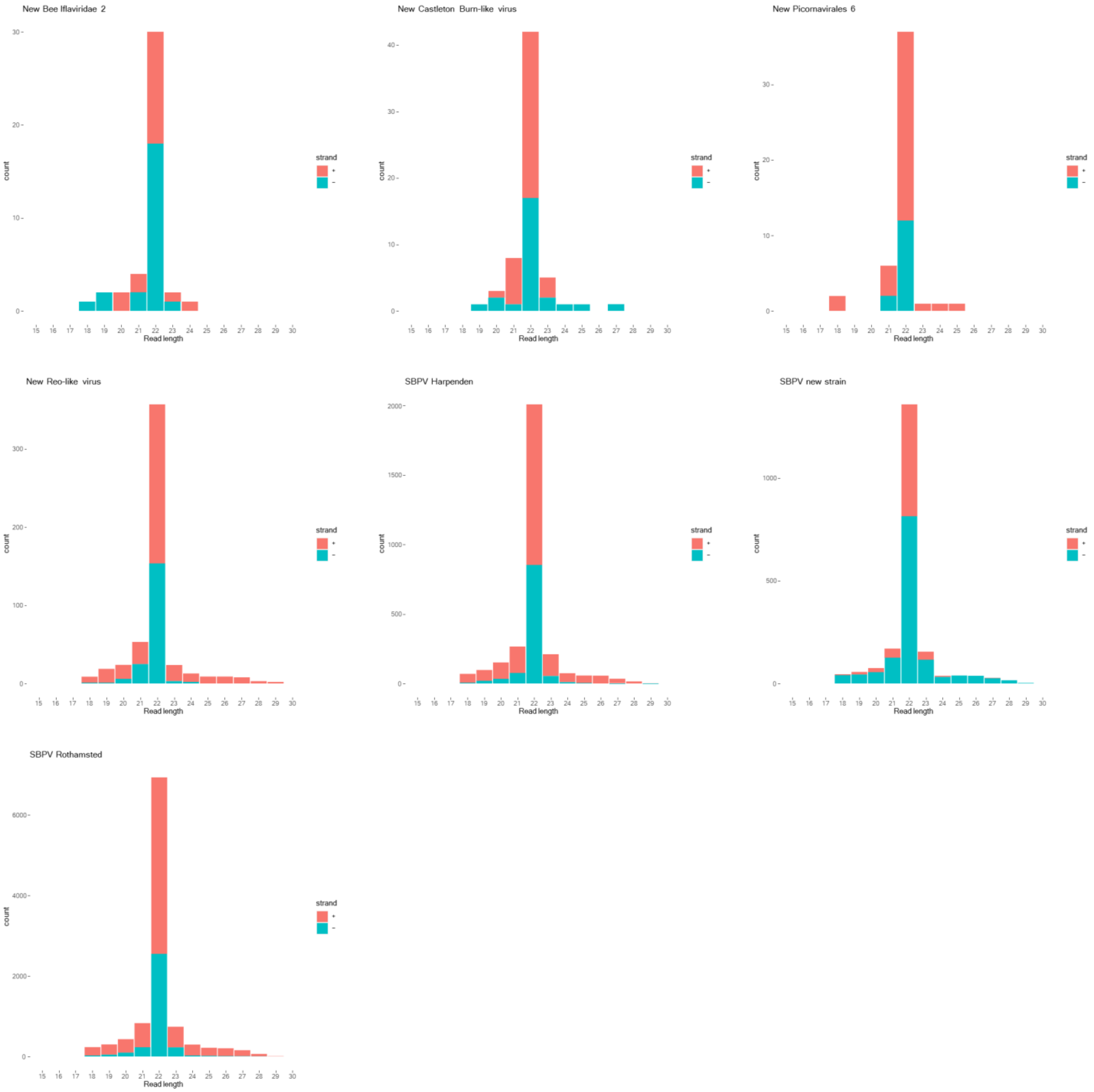

Supplementary Figure 3.36 : *Bombus pascuorum* – June – Plant virus vsiRNA profiles

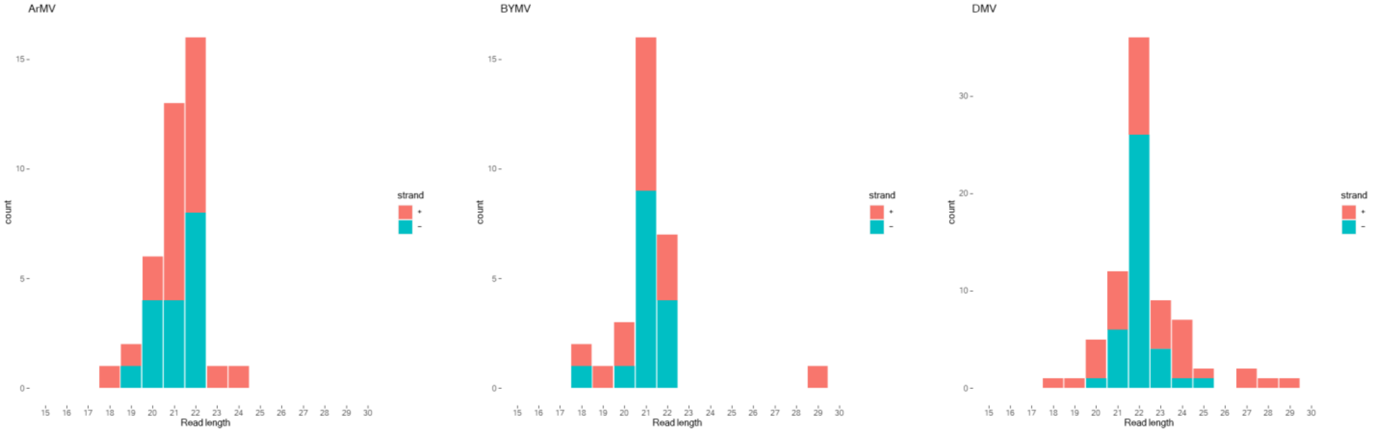

Supplementary Figure 3.37 : *Bombus pascuorum* – June – plant virus vsiRNA profiles

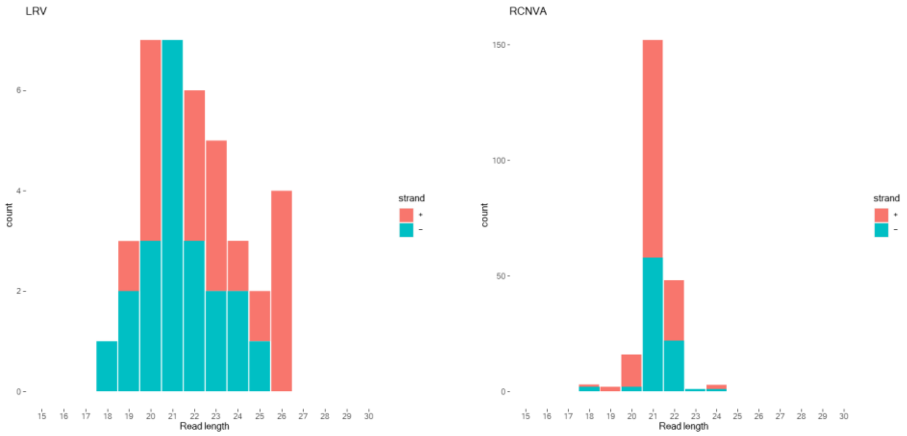

Supplementary Figure 3.38 : *Bombus pascuorum* – August – insect virus vsiRNA profiles

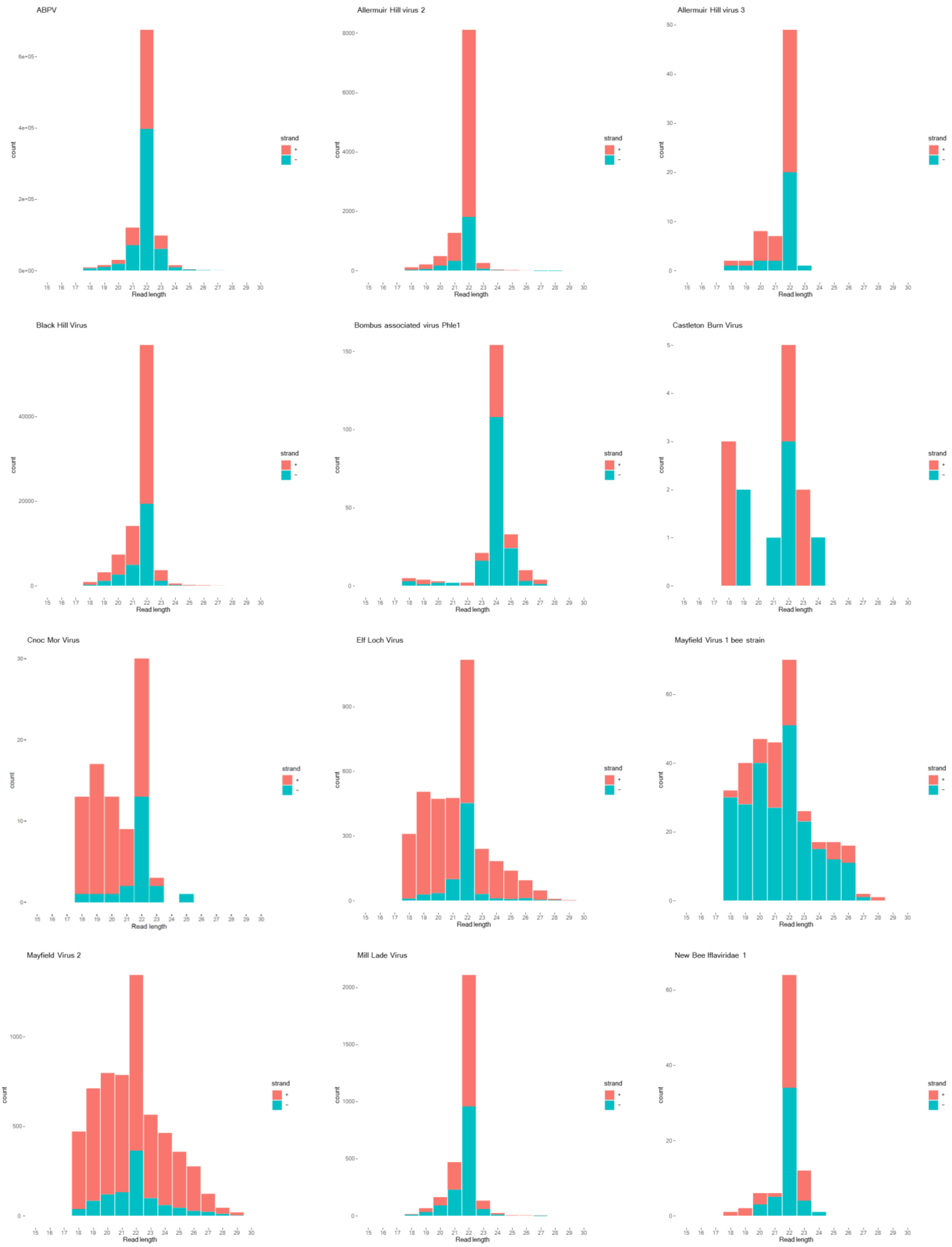

Supplementary Figure 3.39 : *Bombus pascuorum* – August – insect virus vsiRNA profiles

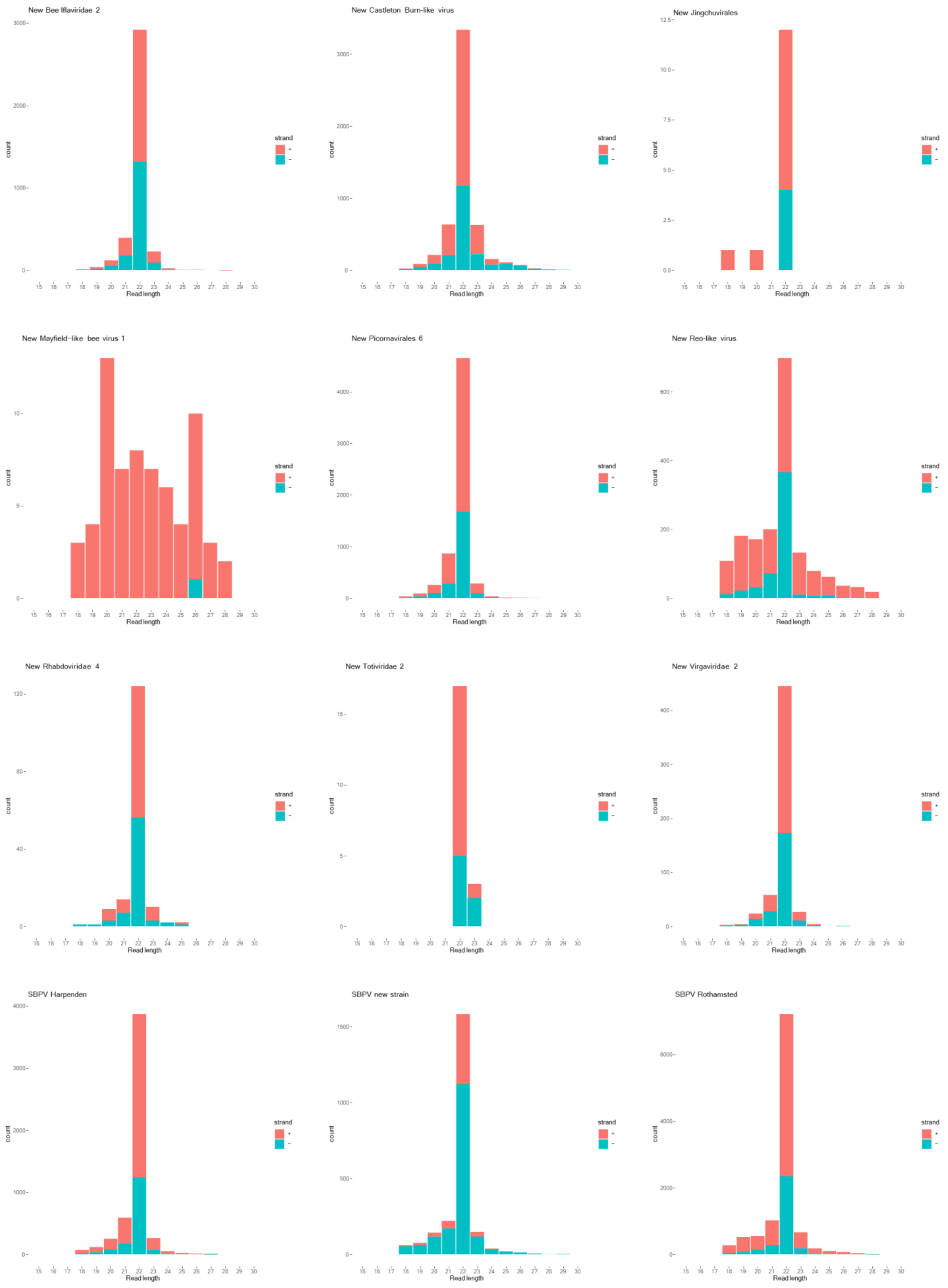

Supplementary Figure 3.40 : *Bombus pascuorum* – August – plant virus vsiRNA profiles

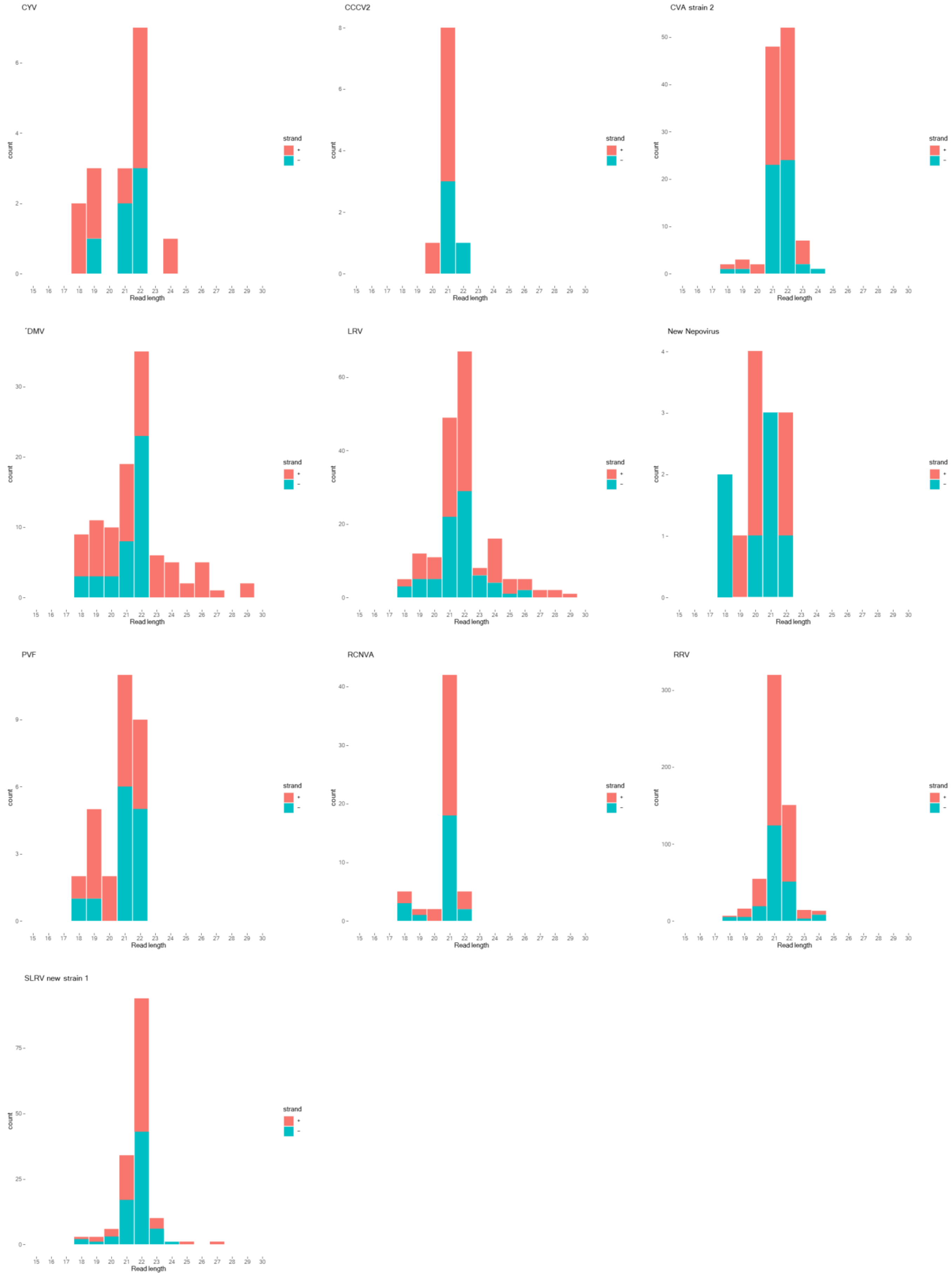

Supplementary Figure 3.41 : *Andrena spp.*– Mixed time points – insect virus vsiRNA profiles

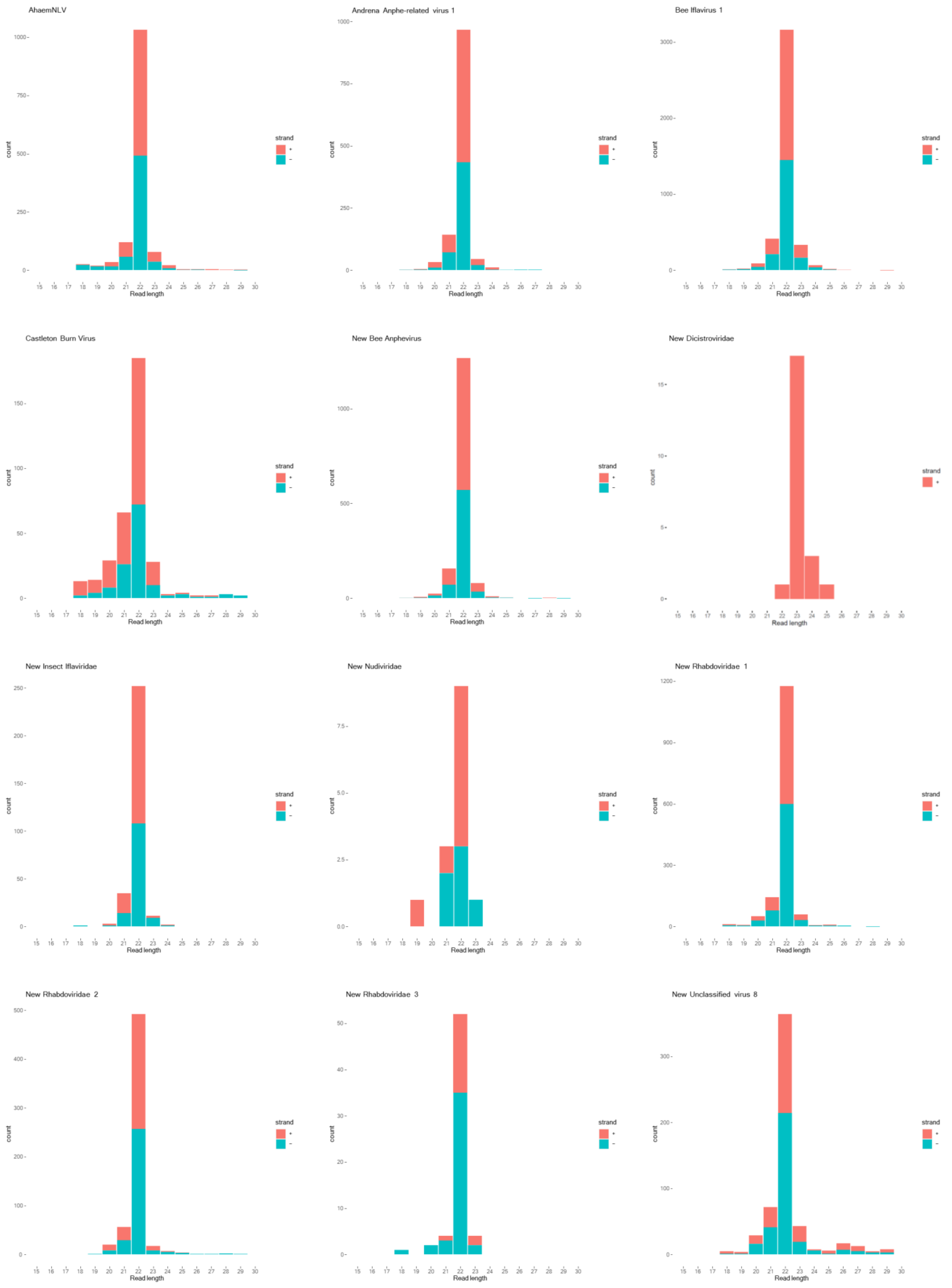

Supplementary Figure 3.42 : *Andrena spp.*– Mixed time points – insect virus vsiRNA profiles

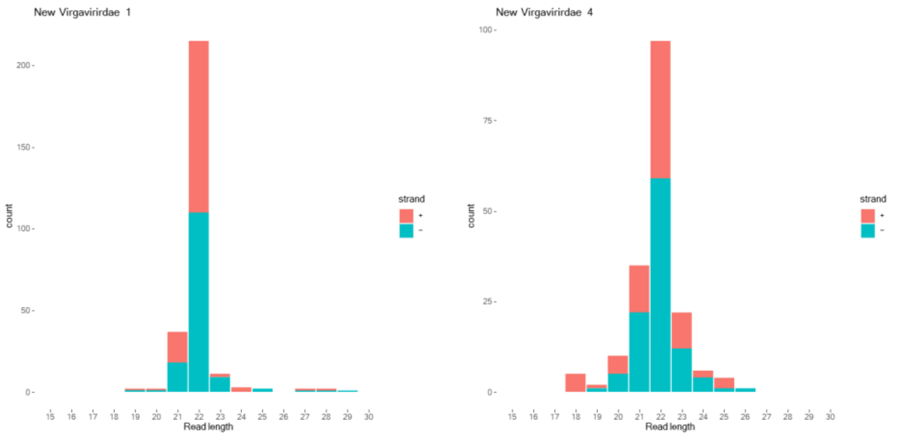

Supplementary Figure 3.43 : *Andrena spp.*– Mixed time points – plant virus vsiRNA profiles

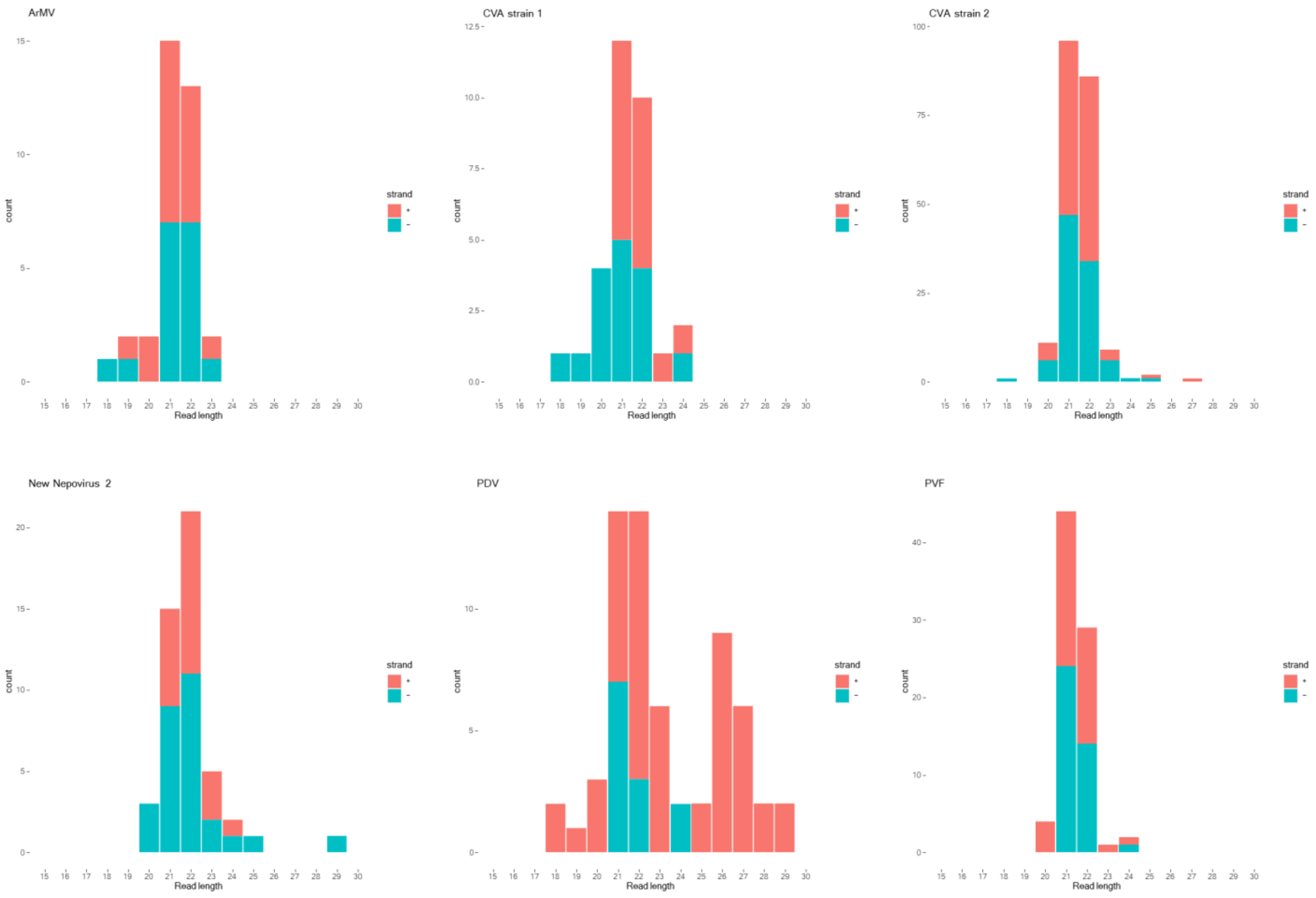

Supplementary Figure 3.44 : Hoverflies – Mixed time points – insect virus vsiRNA profiles

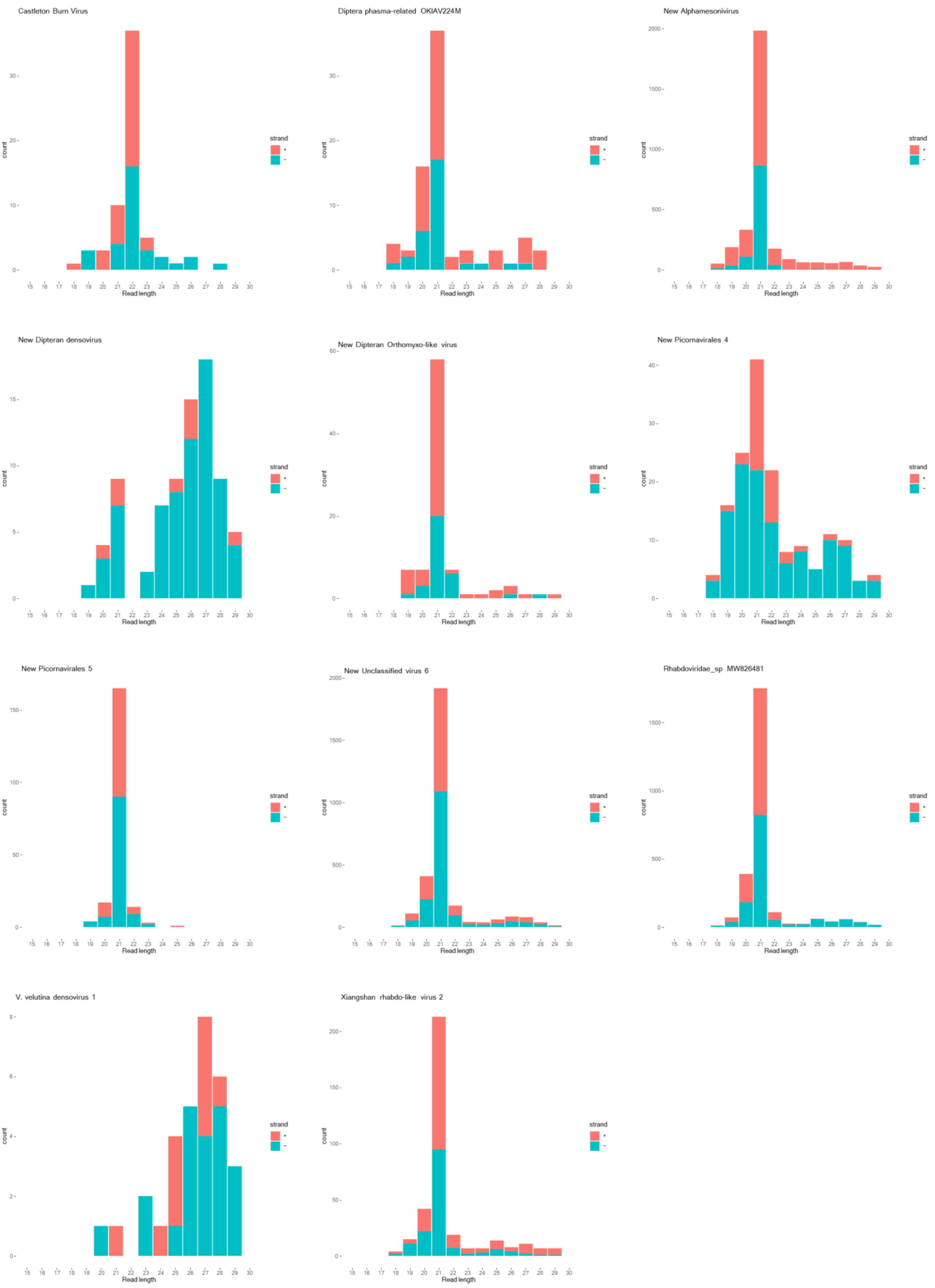

Supplementary Figure 3.45 : Hoverflies – Mixed time points – plant virus vsiRNA profiles

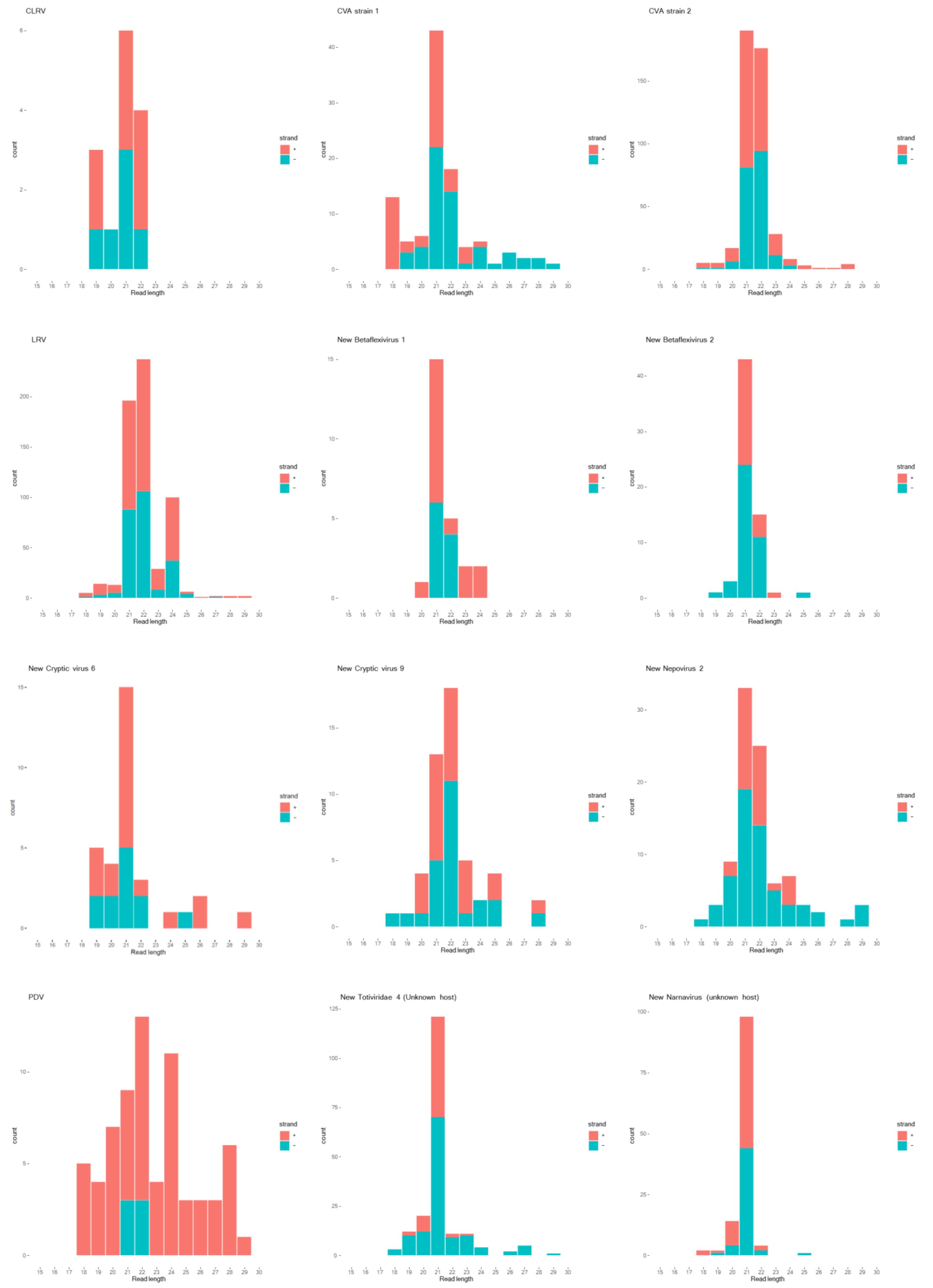

Supplementary Figure 3.46 : Forgotten flies – Mixed time points – insect virus vsiRNA profiles

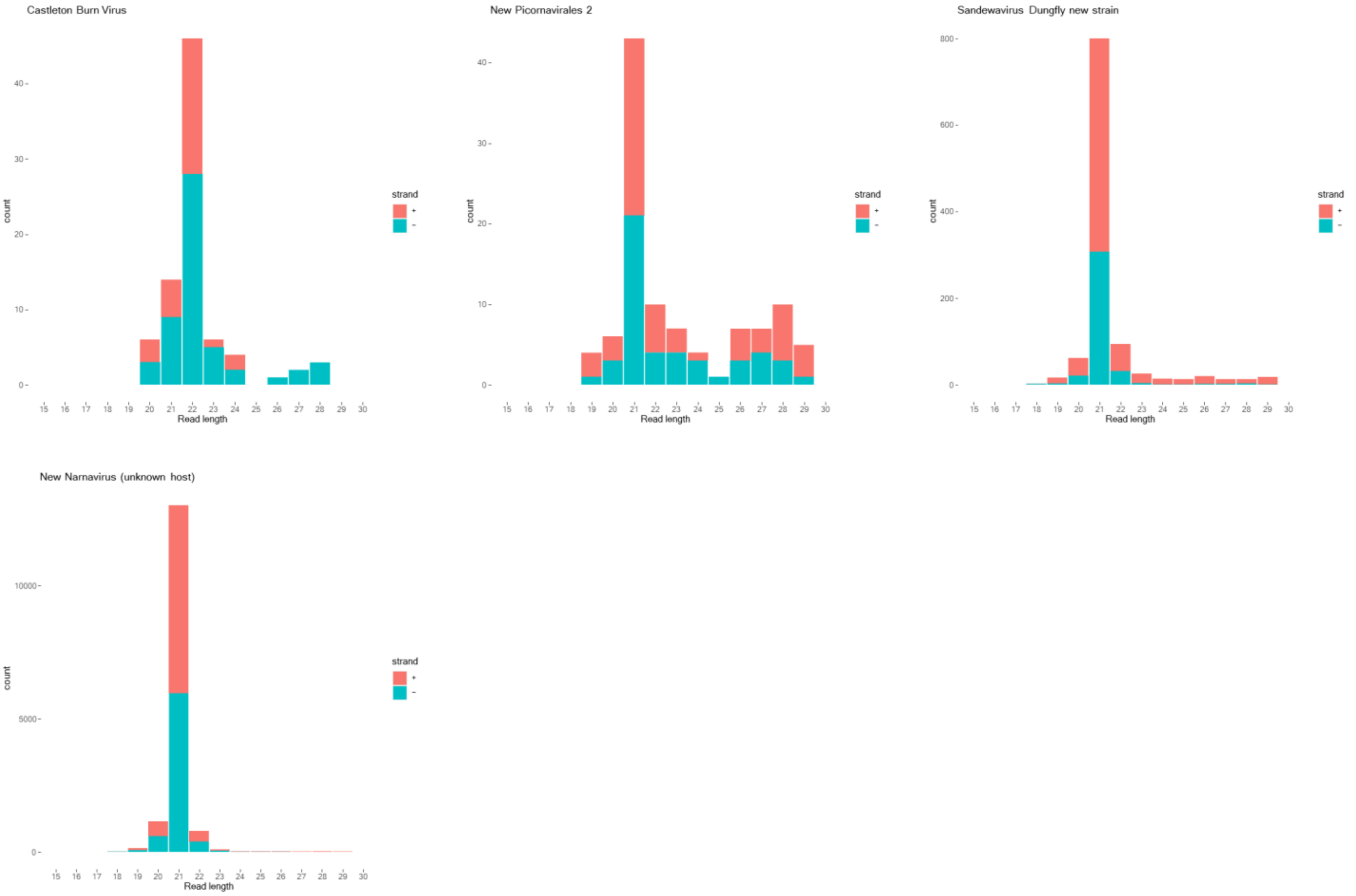

Supplement: Supplementary file 1 — Supplementary Information [file 41467_2025_57314_MOESM1_ESM.pdf]
