# Supplementary material for: Host ecology and phylogeny shape the temporal dynamics of social bee viromes
Source: Nat Commun. 2025 Mar 5;16:2207. doi: 10.1038/s41467-025-57314-7 (PMC11882784; doi:10.1038/s41467-025-57314-7)
Supplement: Supplementary file 2 — Description of Additional Supplementary Files [file 41467_2025_57314_MOESM2_ESM.pdf]

Description of Additional Supplementary Files

Supplementary Data 1: Information about the meta-transcriptome and small RNA libraries, including taxa, collection time, number of pooled samples, BioSample accession numbers (BioProject PRJNA1110080), the total number of reads generated and the proportion of reads mapping to our viral genome targets.

Supplementary Data 2: List of insect viruses identified in meta-transcriptomes and small RNA sequencing from honeybees and bumblebees, used to generate the Venn diagram (Figure 2A).

Supplementary Data 3: Samples dissimilarity indices including Bray-Curtis virome dissimilarity computed for insect viruses and plant viruses separately, COI mitochondrial DNA sequence distance between host species and Horn-Morisita foraging niche dissimilarity measured from plant-pollinator networks.
