# Supplementary material for: Host ecology and phylogeny shape the temporal dynamics of social bee viromes
Source: Nat Commun. 2025 Mar 5;16:2207. doi: 10.1038/s41467-025-57314-7 (PMC11882784; doi:10.1038/s41467-025-57314-7)
Supplement: Supplementary file 6 — Reporting Summary [file 41467_2025_57314_MOESM6_ESM.pdf]

nature portfolio

Corresponding author(s): Vincent Doublet

Last updated by author(s): Feb 4, 2025

Reporting Summary

Nature Portfolio wishes to improve the reproducibility of the work that we publish. This form provides structure for consistency and transparency in reporting. For further information on Nature Portfolio policies, see our Editorial Policies and the Editorial Policy Checklist.

Statistics

For all statistical analyses, confirm that the following items are present in the figure legend, table legend, main text, or Methods section.

n/a	Confirmed	
☐	☒	The exact sample size (*n*) for each experimental group/condition, given as a discrete number and unit of measurement
☐	☒	A statement on whether measurements were taken from distinct samples or whether the same sample was measured repeatedly
☐	☒	The statistical test(s) used AND whether they are one- or two-sided<br>*Only common tests should be described solely by name; describe more complex techniques in the Methods section.*
☐	☒	A description of all covariates tested
☐	☒	A description of any assumptions or corrections, such as tests of normality and adjustment for multiple comparisons
☐	☒	A full description of the statistical parameters including central tendency (e.g. means) or other basic estimates (e.g. regression coefficient) AND variation (e.g. standard deviation) or associated estimates of uncertainty (e.g. confidence intervals)
☐	☒	For null hypothesis testing, the test statistic (e.g. *F*, *t*, *r*) with confidence intervals, effect sizes, degrees of freedom and *P* value noted<br>*Give P values as exact values whenever suitable.*
☒	☐	For Bayesian analysis, information on the choice of priors and Markov chain Monte Carlo settings
☐	☒	For hierarchical and complex designs, identification of the appropriate level for tests and full reporting of outcomes
☒	☐	Estimates of effect sizes (e.g. Cohen's *d*, Pearson's *r*), indicating how they were calculated

Our web collection on statistics for biologists contains articles on many of the points above.

Software and code

Policy information about availability of computer code

Data collection	We did not use any software or code to collect data.
Data analysis	Sequencing data analyses were performed using the following softwares: FastQC (v0.11.8), Sickle (v 1.33), Bowtie2 (v 2.11.0), Samtools (v 1.10), Trinity (v 2.11.0), Blastplus (v 2.11.0), CoverM (v 0.6.1). Statistical analyses were performed with R (v 4.4.2) and using pheatmap (v 1.0.12), vegan (v 2.6-8), bipartite (v 2.20), ggplot2 (v 3.5 1), lme4 (v 1.1-35.5) and blme (v 1.0-6) packages mentioned in the manuscript. No custom code was designed for this study. The scripts used for statistical analyses have been deposited on FigShare (https://doi.org/10.6084/m9.figshare.27888378).

For manuscripts utilizing custom algorithms or software that are central to the research but not yet described in published literature, software must be made available to editors and reviewers. We strongly encourage code deposition in a community repository (e.g. GitHub). See the Nature Portfolio guidelines for submitting code & software for further information.

Data

Policy information about availability of data

All manuscripts must include a data availability statement. This statement should provide the following information, where applicable:

- Accession codes, unique identifiers, or web links for publicly available datasets
- A description of any restrictions on data availability
- For clinical datasets or third party data, please ensure that the statement adheres to our policy

Data Availability:
Meta-transcriptomes and small RNA sequences generated in this study are available in the NCBI SRA database under the BioProject PRJNA1110080 [https://www.ncbi.nlm.nih.gov/bioproject/?term=PRJNA1110080]. Plant pollinator interaction data are available at https://doi.org/10.5061/DRYAD.MSBCC2G2Q87. Source Data for Figures 1, 2 and 3 can be found in Source Data file and Supplementary Data 2 and 3. Viral assemblies and mapping outputs used for Supplementary Figures 2 and 3 are available at https://doi.org/10.6084/m9.figshare.27888378.

Research involving human participants, their data, or biological material

Policy information about studies with human participants or human data. See also policy information about sex, gender (identity/presentation), and sexual orientation and race, ethnicity and racism.

Reporting on sex and gender	No human participants, data or material in this work.
Reporting on race, ethnicity, or other socially relevant groupings	*Please specify the socially constructed or socially relevant categorization variable(s) used in your manuscript and explain why they were used. Please note that such variables should not be used as proxies for other socially constructed/relevant variables (for example, race or ethnicity should not be used as a proxy for socioeconomic status).*<br>*Provide clear definitions of the relevant terms used, how they were provided (by the participants/respondents, the researchers, or third parties), and the method(s) used to classify people into the different categories (e.g. self-report, census or administrative data, social media data, etc.)*<br>*Please provide details about how you controlled for confounding variables in your analyses.*
Population characteristics	*Describe the covariate-relevant population characteristics of the human research participants (e.g. age, genotypic information, past and current diagnosis and treatment categories). If you filled out the behavioural & social sciences study design questions and have nothing to add here, write "See above."*
Recruitment	*Describe how participants were recruited. Outline any potential self-selection bias or other biases that may be present and how these are likely to impact results.*
Ethics oversight	*Identify the organization(s) that approved the study protocol.*

Note that full information on the approval of the study protocol must also be provided in the manuscript.

Field-specific reporting

Please select the one below that is the best fit for your research. If you are not sure, read the appropriate sections before making your selection.

☐ Life sciences ☐ Behavioural & social sciences ☒ Ecological, evolutionary & environmental sciences

For a reference copy of the document with all sections, see nature.com/documents/nr-reporting-summary-flat.pdf

Ecological, evolutionary & environmental sciences study design

All studies must disclose on these points even when the disclosure is negative.

Study description	We collected the most prevalent insect pollinator species from ten farms in Southern England across three time points in one year. We sequenced their meta-transcriptomes to identify and discover RNA viruses. In combination with a temporal analysis of plant-pollinator networks, we tested the effect of host phylogeny and foraging niche on sympatric hosts' viromes.
Research sample	We used meta-transcriptomes of sympatric insect pollinator species, generated from pools of samples following this design: 3 pools (one for April, one for June and one for August) of Apis mellifera, 3 pools for Bombus terrestris, 3 pools for Bombus lapidarius, 2 pools (June and August) for Bombus pascuorum and 2 pools for Bombus hortorum that emerge later in the season, and mixed-species pools from the whole season for less common species: 1 pool of mining bees (Adrena sp.), 1 pool of hoverflies and one pool of other flies named the "Forgotten flies".
Sampling strategy	For each sampling visit, we collected on average 30 of the five most common insect flower visitors based on morpho-groups to obtain reliable prevalence estimates for common pathogens (Manley 2023, https://doi.org/10.1098/rstb.2022.0004). This sampling design was applied on 10 sites, 3 times in one year. Samples were immediately deep frozen in a dry shipper for RNA sequencing and virus analysis.
Data collection	Plant-pollinator interactions were recorded along transects at each farm site and time point. Transects of 100m length and 2 m width

Data collection	were selected based on abundance and richness of flowers and insect visitors within the farm. For each interaction, flower and insect species, or higher taxonomic levels when species could not be determined, were recorded with a pen on a spreadsheet. Data were collected by Vincent Doublet and Toby D. Doyle.
Timing and spatial scale	We visited each farm at three time points to capture the temporal dynamic of viruses in insect pollinators: in spring (12th April to 9th May), in early (18th–30th June) and late (30th July–10th August) summer 2016. To ensure independence of data collection, farms were at least 10 km apart, covering the maximum foraging distance of honeybee workers.
Data exclusions	For the last part of the analysis, where we tested the effect of host phylogeny and foraging niche on sympatric hosts' viromes, we excluded the mixed-species meta-transcriptomes and focussed on viromes sequenced from pools of single species samples.
Reproducibility	Not applicable for this field study. No replication was conducted and samples were pooled per species from all visited field sites to optimize sequencing costs.
Randomization	Randomization was not applicable for this field work (i.e. no treatment involved).
Blinding	Blinding was not applicable for this field study.

Did the study involve field work? ☒ Yes ☐ No

Field work, collection and transport

Field conditions	Samples were collected in farms of Southern England, and the work was performed in favorable conditions only, including wind at a maximum of 5 on the Beaufort scale and a minimum shade temperature of 15 °C in summer and 9°C in spring.
Location	Field sites were located in the South of England, with coordinates provided for the BioProject PRJNA1110080. To preserve anonymity of the involved farmers, no detailed locations are provided.
Access & import/export	Insects were collected from farmland with permission and collaboration with farmers. No further permits were applicable.
Disturbance	Sampling took place on single days for a maximum of ca. 2 hours, i.e. a total of 6 hours per site across 5 months. The impact on population is likely to be minimal, as we collected up to 30 individuals of the 5 most abundant insect pollinator species based on immediately preceding transect walks. The majority of collected specimens are bee workers, i.e. non reproductive animals.

Reporting for specific materials, systems and methods

We require information from authors about some types of materials, experimental systems and methods used in many studies. Here, indicate whether each material, system or method listed is relevant to your study. If you are not sure if a list item applies to your research, read the appropriate section before selecting a response.

Materials & experimental systems

n/a	Involved in the study	
☒	☐	Antibodies
☒	☐	Eukaryotic cell lines
☒	☐	Palaeontology and archaeology
☐	☒	Animals and other organisms
☒	☐	Clinical data
☒	☐	Dual use research of concern
☒	☐	Plants

Methods

n/a	Involved in the study	
☒	☐	ChIP-seq
☒	☐	Flow cytometry
☒	☐	MRI-based neuroimaging

Animals and other research organisms

Policy information about studies involving animals; ARRIVE guidelines recommended for reporting animal research, and Sex and Gender in Research

Laboratory animals	This study does not involve laboratory animals.
Wild animals	For each sampling visit, we collected on average 30 of the five most common insect flower visitors based on morpho-groups. Insect were caught on the wing and immediately stored in a clean sampling vial, in the dark in a cool bag until samples were deep frozen in a dry shipper for RNA sequencing and virus analysis. All insects were adults of unknown age. Collected species were: Apis mellifera, Bombus terrestris, Bombus lapidarius, Bombus hortorum, Bombus pascuorum, mining bees (Adrena sp.), and mixed fly (Diptera) species.
Reporting on sex	Viromes were sequenced from a mix of males and females, except for honeybees (males do not forage). Because samples were

Reporting on sex | pooled, sex was not considered as a variable in this study.

Field-collected samples | Field-collected samples were bisected frozen and we subsequently proceeded to RNA isolation.

Ethics oversight | Ethics oversight was by the University of Exeter, and handled by the CLES Penryn Ethics Committee.

Note that full information on the approval of the study protocol must also be provided in the manuscript.

Plants

Seed stocks | No plant work in this study.

Novel plant genotypes | *Describe the methods by which all novel plant genotypes were produced. This includes those generated by transgenic approaches, gene editing, chemical/radiation-based mutagenesis and hybridization. For transgenic lines, describe the transformation method, the number of independent lines analyzed and the generation upon which experiments were performed. For gene-edited lines, describe the editor used, the endogenous sequence targeted for editing, the targeting guide RNA sequence (if applicable) and how the editor was applied.*

Authentication | *Describe any authentication procedures for each seed stock used or novel genotype generated. Describe any experiments used to assess the effect of a mutation and, where applicable, how potential secondary effects (e.g. second site T-DNA insertions, mosiacism, off-target gene editing) were examined.*
